# Structural basis for differential recognition of phosphohistidine-containing peptides by 1-pHis and 3-pHis monoclonal antibodies

**DOI:** 10.1073/pnas.2010644118

**Published:** 2021-02-05

**Authors:** Rajasree Kalagiri, Robyn L. Stanfield, Jill Meisenhelder, James J. La Clair, Stephen R. Fuhs, Ian A. Wilson, Tony Hunter

**Affiliations:** ^a^Molecular and Cell Biology Laboratory, Salk Institute for Biological Studies, La Jolla, CA 92037;; ^b^Department of Integrative Structural and Computational Biology, The Scripps Research Institute, La Jolla, CA 92037;; ^c^Department of Chemistry and Biochemistry, University of California San Diego, La Jolla, CA 92093;; ^d^Jack H. Skirball Center for Chemical Biology and Proteomics, Salk Institute for Biological Studies, La Jolla, CA 92037;; ^e^The Skaggs Institute for Chemical Biology, The Scripps Research Institute, La Jolla, CA 92037

**Keywords:** phosphohistidine antibodies, histidine phosphorylation, posttranslational modifications

## Abstract

In 2015, monoclonal antibodies (mAbs) that selectively recognize the 1-pHis or 3-pHis isoforms of phosphohistidine were developed by immunizing rabbits with degenerate Ala/Gly peptides containing the nonhydrolyzable phosphohistidine (pHis) analog- phosphotriazolylalanine (pTza). Here, we report structures of five rabbit mAbs bound to cognate pTza peptides: SC1-1 and SC50-3 that recognize 1-pHis, and their 3-pHis–specific counterparts, SC39-4, SC44-8, and SC56-2. These cocrystal structures provide insights into the binding modes of the pTza phosphate group that are distinct for the 1- and 3-pHis mAbs with the selectivity arising from specific contacts with the phosphate group and triazolyl ring. The mode of phosphate recognition in the 3-pHis mAbs recapitulates the Walker A motif, as present in kinases. The complementarity-determining regions (CDRs) of four of the Fabs interact with the peptide backbone rather than peptide side chains, thus conferring sequence independence, whereas SC44-8 shows a proclivity for binding a GpHAGA motif mediated by a sterically complementary CDRL3 loop. Specific hydrogen bonding with the triazolyl ring precludes recognition of pTyr and other phosphoamino acids by these mAbs. Kinetic binding experiments reveal that the affinity of pHis mAbs for pHis and pTza peptides is submicromolar. Bound pHis mAbs also shield the pHis peptides from rapid dephosphorylation. The epitope–paratope interactions illustrate how these anti-pHis antibodies are useful for a wide range of research techniques and this structural information can be utilized to improve the specificity and affinity of these antibodies toward a variety of pHis substrates to understand the role of histidine phosphorylation in healthy and diseased states.

## Significance

Phosphohistidine (pHis) is a labile posttranslational modification with two isoforms, 1-pHis and 3-pHis, involved in many cellular processes across the kingdoms of life. Due to its lability, it is difficult to study the pHis modification using standard biochemical tools and techniques. Previously, we developed monoclonal antibodies (mAbs) against the 1-pHis and 3-pHis modifications using stable phosphotriazolylalanine mimetics as immunogens. These antibodies are promising tools to uncover the role of pHis in eukaryotic cells. Here, we report the crystal structures of five of these mAbs bound to their cognate phosphotriazolylalanine peptides, thus providing insight into the structure–function relationships that guide pHis recognition and establishing a foundation for the structure-guided design of improved pHis antibodies.

Phosphorylation is a crucial posttranslational modification that extends the functionality and versatility of the cellular proteome (1). Of the nine amino acids that can undergo O/N/S-phosphorylation on their side chains, pSer/pThr and pTyr are readily studied due to the chemical stability of their phosphomonoester linkages (O-P) and, as a result have been implicated in many cellular processes and diseases (2). On the other hand, histidine undergoes N-linked phosphorylation on either nitrogen on its imidazole ring to form a high-energy phosphoramidate bond (N-P), which is labile at low pH and high temperature (3). Histidine is the only amino acid that can undergo asymmetric phosphorylation on its side chain, thus giving rise to two isoforms or positional isomers, 1-phosphohistidine (1-pHis) and 3-phosphohistidine (3-pHis). The position of phosphate moiety at the first or π (pros) or third or τ (tele) position on the imidazole side chain exhibits different kinetic and thermodynamic properties. The N-P bond stability of 1-pHis and 3-pHis depends on pH with 1-pHis being relatively unstable below pH 7 when compared to 3-pHis (4). X-ray crystallography and NMR studies have revealed that the 1- or 3-pHis modifications of proteins are mutually exclusive. For example, autophosphorylated nucleoside diphosphate kinase family phosphoenzyme intermediates have only the 1-pHis modification (5), whereas succinyl Co-A synthetase (SCS) (6), phosphoglycerate mutase 1 (7), and phosphofructokinase bisphosphatase (8) phosphoenzyme intermediates exhibit the 3-pHis modifications. Understanding the role and mechanisms guiding this selectivity are vital to develop a mechanistic understanding of His phosphorylation.

Since its discovery in the 1960s, His phosphorylation has been revealed as a ubiquitous player both in prokaryotes and eukaryotes (9, 10). Two-component systems in bacteria, fungi, and plants use His phosphorylation in coupling environmental signals to the cellular outcomes that include virulence, survival, and quorum sensing (11–13). His phosphorylation in eukaryotes also plays a vital role in regulating cellular processes, such as nucleotide homeostasis, ion channel regulation, or G protein signaling (14–16). Despite being difficult to study due to its lability, the pHis modification and its roles in prokaryotic and eukaryotic biology have been studied using thin-layer chromatography, high-pressure liquid chromatography, NMR spectroscopy, and mass spectrometry (MS). Recently, phosphoproteomic analysis using TiO_2_ metal oxide affinity chromatography of tryptic peptides obtained from zebrafish larvae suggested that 6% of global protein phosphorylation is contributed by pHis (17). Similarly, 12% of bacterial phosphorylation is attributed to pHis (18). The percentage of pHis uncovered in these studies is relatively more than that of the well-studied pTyr modification (8%). These findings make it all the more important to understand the role of His phosphorylation in both prokaryotes and eukaryotes. Development of reagents that are specific for pHis modifications are needed to validate the large-scale phosphoproteomic substrate identifications and explore functional significance of pHis modifications.

It has been challenging to develop antibodies specific for pHis due to the rapid hydrolysis of the phosphoramidate bond and the spontaneous isomerization of 1-pHis to the more stable 3-pHis (19). Hence, efforts have been directed to develop synthetic pHis mimetics that are nonhydrolyzable and nonisomerizable that can be used for immunizations. Sequence-specific and pan-pHis antibodies were developed previously, using the pHis mimetics pTza (phosphotriazolylalanine), pTze (phosphoryltriazolylethylamine), and pPye (phosphonopyrazolylethylamine), where the phosphoramidate bond (N-P) is replaced by the more stable phosphonate linkage (C-P). However, these antibodies suffered from low affinity and significant cross-reactivity with proteins containing pTyr modifications (20–22). In 2015, we reported the development of rabbit monoclonal antibodies (mAbs) against each of the two pHis isomers using stable analogs, 1- and 3-phosphotriazolylalanine (pTza) embedded in a degenerate peptide sequence of Ala and Gly (23). These mAbs were shown to be sequence independent and lack cross-reactivity to the other pHis isoform and the pTyr modification. These mAbs were used in cellular assays like immunofluorescence staining, which revealed that 1-pHis might play a role in phagocytosis and 3-pHis in mitosis. These antibodies could also be used in immunoaffinity purification and MS analysis to identify potential pHis substrates in a cellular context (23). To rationalize their properties, we set out to define the structures of these mAbs bound to pTza-containing peptides using macromolecular crystallography.

Here, we report structures of Fab fragments derived from five rabbit pHis mAbs crystallized in complex with their cognate 1-pTza and 3-pTza peptides. These structures provide critical information about the molecular mechanism of phosphoepitope recognition, isomer specificity, sequence independence, and noncross-reactivity to other phosphoamino acids. Thermal stability assays and kinetic binding assays with pHis peptides reveal that these antibodies do not differentiate the pHis mimetic pTza from the native pHis modification in terms of recognition. The structural information presented here provides a guide for design of next-generation antibodies with increased affinity and specificity which can be applied quantitatively and qualitatively to a wide array of substrates, thereby advancing our understanding of pHis biology.

## Results

### Structure of the pHis mAbs.

**Fig. 1.**
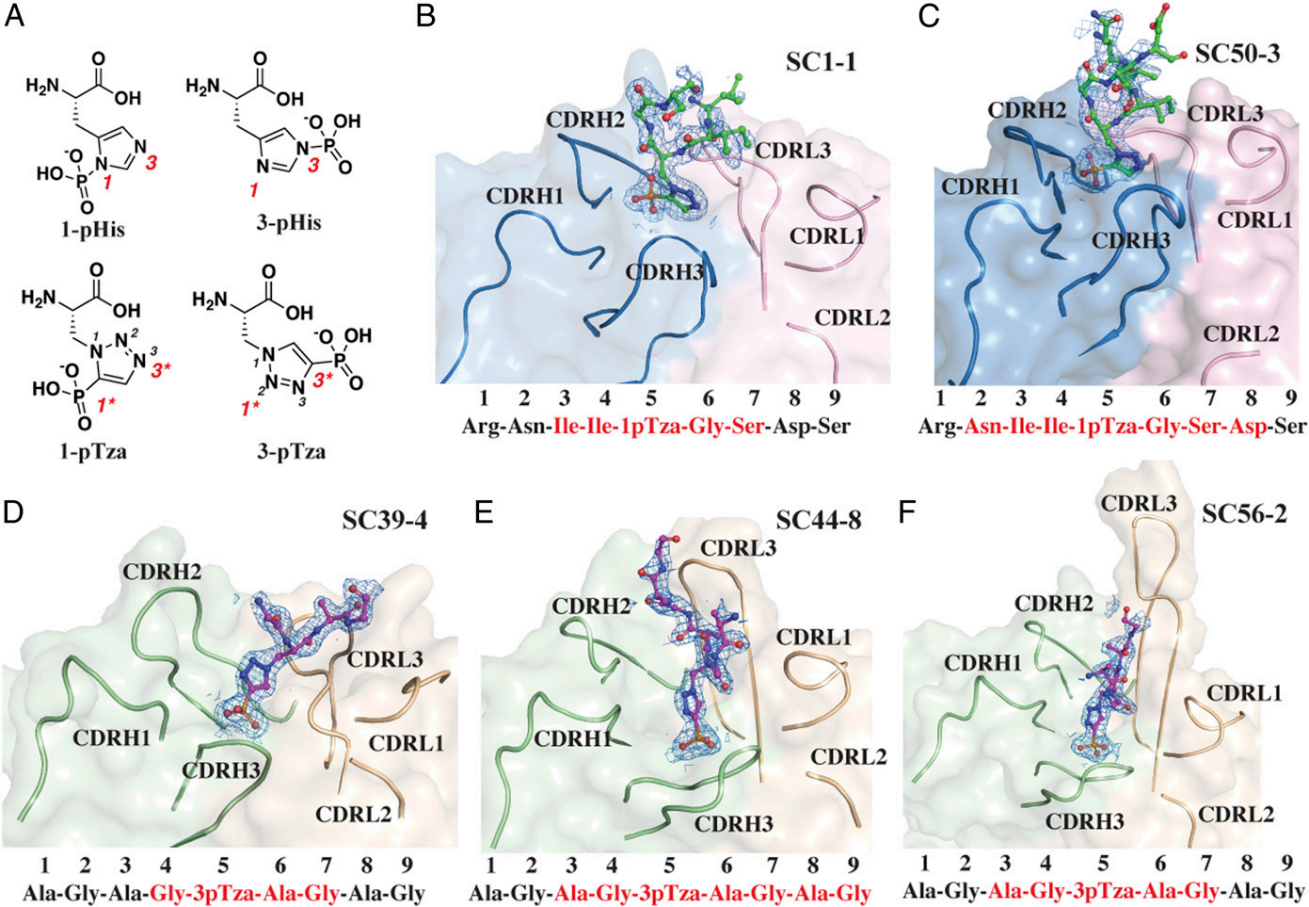
Structures of pHis Fabs bound to pTza peptides. (*A*) The structures of isomers of phosphohistidine (pHis) 1-pHis and 3-pHis and, phosphotriazolylalanine (pTza) 1-pTza and 3-pTza. Nitrogens that undergo phosphorylation are numbered (red font) on pHis isomers and the corresponding positions are numbered and starred (red font) on pTza isomers. Crystal structures of pHis Fabs, SC1-1 (*B*) and SC50-3 (*C*) (blue: heavy chain; pink: light chain; and green: peptide) bound to NM23-1-pTza peptide and, SC39-4 (*D*), SC44-8 (*E*), and SC56-2 (*F*) (light green: heavy chain; wheat: light chain; and magenta: peptide) bound to ACLYana-3-pTza peptide. *B*–*F* also show the omit map (*F*_*O*_*–F*_*C*_ map) for the peptide contoured at 1.0 σ of the peptide (NM23-1-pTza and ACLYana-3-pTza) bound in the antibody combining site. The sequence of the peptide used for crystallization is shown below *B*–*F* and red font indicates the peptide residues with interpretable electron density.

As an approach to understand the interaction of anti-pHis mAbs with pHis substrates in atomic detail, we solved the cocrystal structures of five different pHis Fabs bound to their cognate pTza peptides using X-ray crystallography (*SI Appendix*, Table S1). As authentic 1-pHis and 3-pHis peptides would likely be hydrolyzed during crystallization, peptides containing the nonhydrolyzable pHis mimetics, 1- and 3-pTza (phosphotriazolylalanine) were synthesized and used for cocrystallization with pHis Fabs (Fig. 1*A* and *SI Appendix*, Table S2). The 1-pHis Fabs, SC1-1 and SC50-3, were cocrystallized with a 9-amino acid NM23-1-pTza peptide (RNII-1-pTza-GSDS), corresponding to the sequence around the active site pH118 residue on NM23 (*SI Appendix*, Table S2). The expressed SC1-1 and the SC50-3 Fabs share the same SC1-1 light chain, with significant homology (82% identity) also present in the heavy-chain complementarity-determining regions (CDRs) (*SI Appendix*, Fig. S1). SC1-1 has four molecules in the asymmetric unit with RMSD between 0.3 Å and 1.1 Å, whereas SC50-3 has RMSD between 0.24 Å and 1.07 Å among six molecules of the asymmetric unit. The molecule with the best electron density was chosen for description in the sections below. The cocrystal structure of SC1-1:NM23-1-pTza exhibited ordered electron density only for residues II-1-pTza-GS, whereas in the structure for the SC50-3:NM23-1-pTza complex, clear density was visualized for residues NII-1-pTza-GSD. The bound peptides adopt a type I β-turn conformation around residues I-1-pTza-GS (Fig. 1 *B* and *C*). The heavy chain of SC50-3 in the SC50-3:NM23-1-pTza is glycosylated at residue Asn97 with ordered electron density for up to two *N*-acetylglucosamine and one fucose moieties. The two Fabs (variable and constant domains) superpose with an RMSD of 1.3 Å on the Cα carbons of the framework region of the antibody and with an RMSD of 1.8 Å for their CDRs and, have an elbow angle between 146° and 159° for different protomers in the asymmetric units of SC1-1 and SC50-3 (*SI Appendix*, Table S3), as measured by the RBOW server (24). The peptides in the two 1-pHis antibody complexes (SC1-1:NM23-1-pTza and SC50-3:NM23-1-pTza) have a Cα RMSD of 1.4 Å with minimum changes at the bound pTza and peptide (*SI Appendix*, Fig. S2*A*). Around 304 Å^2^ and 290 Å^2^ of molecular surface are buried on the Fab and peptide in SC1-1:NM23-1-pTza, respectively, and 347 Å^2^ (Fab) and 288 Å^2^ (peptide) in the SC50-3:NM23-1-pTza (*SI Appendix*, Table S4). In both cases, the heavy chain provides the majority of interactions with the pTza moiety, whereas the light chain contributes to peptide binding.

The three 3-pHis antibodies—SC39-4, SC44-8, and SC56-2—were cocrystallized with the nonapeptide ACLYana-3-pTza (AGAG-3-pTza-AGAG) (*SI Appendix*, Table S2). SC39-4 has two molecules in the asymmetric unit with 0.12 Å RMSD. In the crystal structure of SC39-4:ACLYana-3-pTza, ordered electron density was seen for four residues (G-3-pTza-AG), whereas SC44-8:ACLYana-3-pTza had interpretable density for seven residues (AG-3-pTza-AGAG), and SC56-2:ACLYana-3-pTza for five residues (AG-3-pTza-AG) (Fig. 1 *D*–*F*). These mAbs have high sequence variability in the CDRH2, CDRH3, CDRL1, and CDRL3, but low variability in CDRH1 and CDRL2 (*SI Appendix*, Fig. S1). The lengths of the CDRL3 are also different in these antibodies with a range of 13 to 19 amino acids, typical of rabbit mAbs (25). The peptide overlay of three 3-pHis antibody:peptide complexes shows that the phosphate binding is more conserved, with very low RMSDs, whereas the peptide has high degrees of freedom in binding (*SI Appendix*, Fig. S2*B*). The elbow angles of the 3-pHis Fabs are between 142° and 150° (*SI Appendix*, Table S3), as measured by the RBOW server (24). The SC39-4:ACLYana-3-pTza interaction buries 230 Å^2^ and 216 Å^2^ of molecular surface on Fab and peptide, respectively. In SC44-8:ACLYana-3-pTza, 384 Å^2^ (Fab) and 372 Å^2^ (peptide) are buried and in SC56-2:ACLYana-3-pTza, 281 Å^2^ (Fab) and 254 Å^2^ (peptide) (*SI Appendix*, Table S4). In SC39-4:ACLYana-3-pTza, the light chain contributes to binding the pTza moiety more than the heavy chain while for SC44-8:ACLYana-3-pTza and SC56-2:ACLYana-3-pTza, heavy and light chains contribute equally to binding to the pTza group. The peptide interactions with these antibodies are mediated predominantly by the light chain. All the pHis Fabs have a K1 type light chain, which contains one atypical intrachain disulfide bond between Cys80 (variable domain) and Cys171 (constant domains) (26).

### Structural Basis for Phosphate Recognition.

A common feature that pHis Fabs share with the conventional phosphate-binding structural scaffolds such as the P-loop or Walker A motif is the positive electrostatic potential lining the antibody groove in the phosphate-binding region (*SI Appendix*, Fig. S3) (27). This positively charged surface plays a significant role in guiding the seating of the negatively charged phosphate group in the binding pocket with minimum repulsions. The major differences between the phosphate recognition by the 1-pHis and 3-pHis Fabs are the mode of binding, wherein different sets of residues play a key role in orientation of the phosphate moiety of the peptide within in the CDR region.

**Fig. 2.**
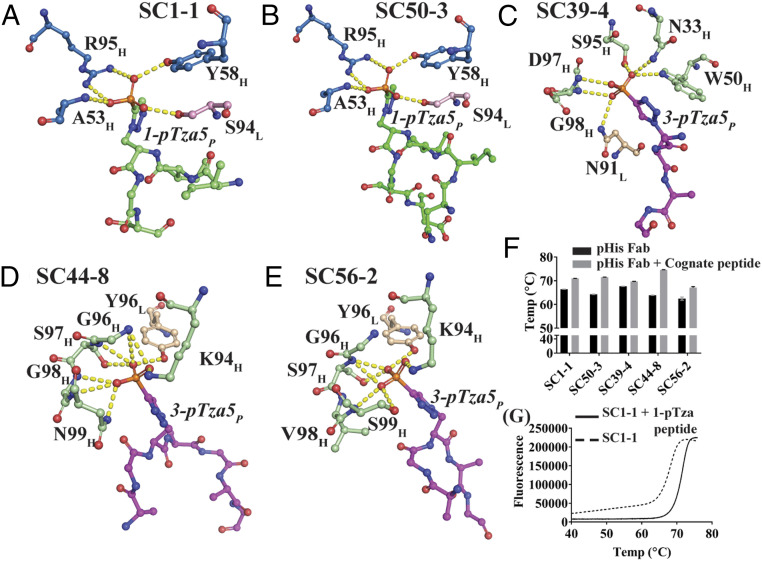
Phosphate versus nonphosphate recognition of pHis Fabs. Snapshots of the atomic interactions of the peptide phosphate moiety with the interacting residues from the CDR regions of pHis Fabs. Most of the interactions are through hydrogen bonds (<3.9 Å) along with a few salt bridges (*SI Appendix*, Table S9). For SC1-1 (*A*) and SC50-3 (*B*) (blue: heavy chain; pink: light chain; and green: peptide), the major contribution to the binding is by the CDRH2 loop with additional interactions from CDRH3 and CDRL3. SC39-4 (*C*), SC44-8 (*D*), and SC56-2 (*E*) (light green: heavy chain; wheat: light chain; and magenta: peptide) makes interactions with CDRH3 with additional interactions from CDRL3; CDRH1 and CDRH2 are also used in SC39-4. (*F*) The DSF analysis of pHis Fabs in presence of their cognate pTza peptides shows that the *T*_m_ of the antibodies increases in the presence of phosphorylated peptide indicating binding of the pTza peptide. (*G*) A representative thermal melt plot of SC1-1 in presence and absence of ACLYana-1-pTza peptide (cognate).

In the 1-pHis Fabs the peptidyl phosphate is oriented toward the CDRH2 loop, whereas it is anchored on CDRH3 in the 3-pHis antibodies thus creating a deeper pocket (Fig. 1). In the SC1-1:NM23-1-pTza, the peptidyl phosphate group forms hydrogen bonds with the main-chain amide of Ala53 and the side-chain hydroxyl group of Tyr58 in CDRH2. Arg95 in CDRH3 undergoes a bidentate salt bridge with the phosphate group. An additional hydrogen bond is contributed by the hydroxyl group of Ser94 in the CDRL3 loop (Fig. 2*A*). The auxiliary interactions are mediated by two water molecules sequestered in the binding site (*SI Appendix*, Fig. S4*A*). The SC50-3:NM23-1-pTza (2.34 Å) is similar to SC1-1:NM23-1-pTza (1.65 Å) with the same residues interacting with the peptidyl phosphate; however, it only contains a single water-mediated interaction (Fig. 2*B* and *SI Appendix*, Fig. S4*B*). Whether loss of this water molecule is real or a function of the resolution is not known.

In the SC39-4:ACLYana-3-pTza, Asn33 in CDRH1, Trp50 in CDRH2, Ser95, Asp97, and Gly98 in CDRH3, and Asn91 in CDRL3 display hydrogen bonds with the peptidyl phosphate group (Fig. 2*C*). Alternatively, in the SC44-8:ACLYana-3-pTza and SC56-2:ACLYana-3-pTza complexes, interactions with the peptidyl phosphate occurs only through CDRH3 and CDRL3, unlike SC39-4:ACLYana-3-pTza. In the SC44-8:ACLYana-3-pTza, Lys94, Gly96, Ser97, Gly98, and Asn99 in the CDRH3 loop form a concave pocket to support phosphate binding. It is further supported by Tyr96 within CDRL3 (Fig. 2*D*). The SC56-2:ACLYana-3-pTza shares the same phosphate-binding residues with SC44-8:ACLYana-3-pTza peptide complex except for substitution with Val98 and Ser99 (Fig. 2*E*). The SC39-4:ACLYana-3-pTza maintains additional hydrogen bonding with peptidyl phosphate through two water molecules that mediate interaction with residues in the CDRH3 and CDRL3 loops (*SI Appendix*, Fig. S4*C*), whereas both the SC44-8:ACLYana-3-pTza and the SC56-2:ACLYana-3-pTza complexes have one conserved water molecule that mediates interaction of phosphate with CDRH1 and CDRH2 residues (*SI Appendix*, Fig. S4 *D* and *E*). The binding energy contributed by the five to nine hydrogen bonds (*SI Appendix*, Table S5) with the peptidyl phosphate group is the key for differentiation of phosphorylated versus nonphosphorylated substrates by pHis mAbs (*SI Appendix*, Fig. S5).

Next, we examined the ability of these Fabs to recognize phosphorylated substrates using differential scanning fluorimetry (DSF). The thermal melting temperatures of the pHis Fabs were significantly increased by 2 to 7 °C in presence of the cognate ACLYana-1-pTza or ACLYana-3-pTza peptides when compared to unliganded Fabs (Fig. 2 *F* and *G*). In concert, pHis mAbs slowed the dephosphorylation of bound pHis peptide (ACLYana-pHis) by sequestering the phosphoramidate linkage of the peptide from the solvent (*SI Appendix*, Fig. S6).

### Comparative Kinetic Analyses of pHis Fabs with pTza Peptides versus pHis Peptides.

**Fig. 3.**
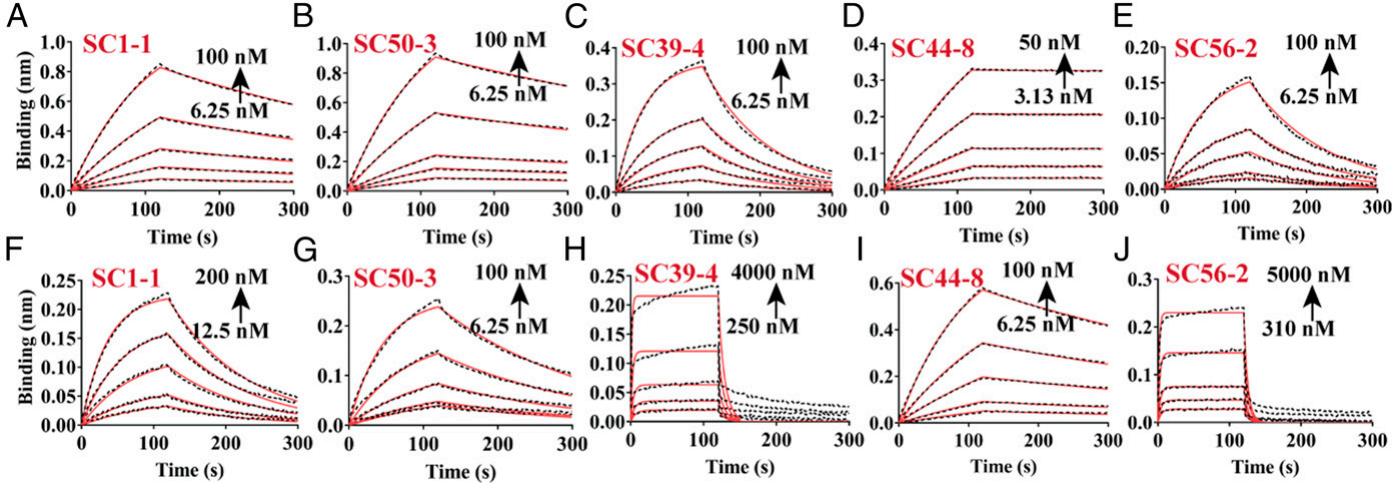
Binding kinetics of pHis Fabs with cognate pTza and pHis peptides. BLI studies of immobilized peptides (ACLYana-1/3-pTza and phosphorylated ACLYana) with varying concentrations of the pHis Fabs were carried out in triplicate on an Octet Red96e instrument (FortéBio). SC1-1 (*A*) and SC50-3 (*B*) pHis Fab interaction with their ACLYana-1-pTza peptides. SC39-4 (*C*), SC44-8 (*D*), and SC56-2 (*E*) pHis Fabs interactions with ACLYana-3-pTza peptides. SC1-1 (*F*), SC50-3 (*G*), SC39-4 (*H*), SC44-8 (*I*), and SC56-2 (*J*) pHis Fabs interactions with their affinity-purified ACLYana-pHis peptides are represented, *Right*. The experimental data are represented in the black dashed lines and the nonlinear least-squares fitting was represented in the red continuous lines. The χ^2^ for the goodness of fit is under 1.0 for the all the plots. The rate constants and affinity values are provided in Table 1.

**Fig. 4.**
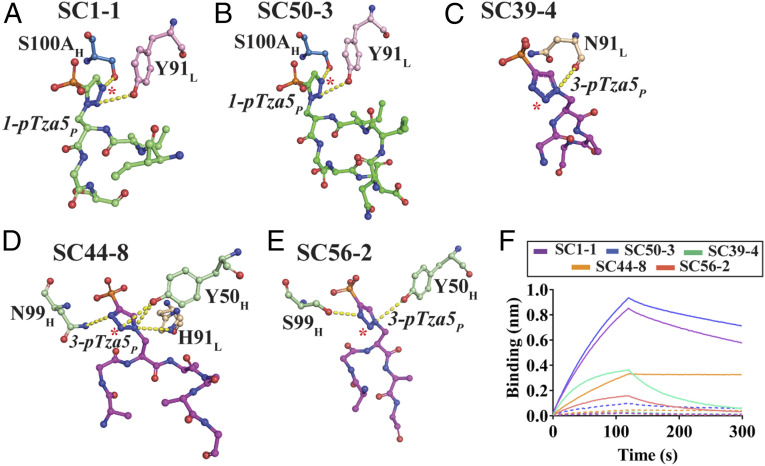
Isomer specificity of pHis Fabs. The hydrogen bond interactions of SC1-1 (*A*), SC50-3 (*B*), SC39-4 (*C*), SC44-8 (*D*), and SC56-2 (*E*) with the triazolyl group (analogous to imidazole group on Histidine) of pTza moiety. SC1-1 and SC50-3 use CDRH3 and CDRL3 to bind the triazolyl group, whereas SC39-4 uses CDRL3, SC44-8 and SC56-2 use CDRH2 and CDRH3. The atoms on triazolyl group corresponding to nitrogen on the imidazole, which are free to undergo hydrogen bonding, are starred. These interactions may form when pHis mAbs bind to pHis peptides. (*F*) Interferometry studies of pHis Fabs in presence of cognate (continuous lines) versus noncognate peptides (dashed lines) indicate that the Fabs bind to cognate peptides with greater affinity than to noncognate peptides.

Biolayer interferometry (BLI) studies were performed to determine the relative affinity of pHis Fabs to in situ phosphorylated His peptides versus the phosphomimetic substrates. The N-terminally biotinylated peptides were immobilized on streptavidin biosensors and the rate of association (*k*_*a*_) and dissociation (*k*_*d*_) were determined by varying the pHis Fab concentration. Equilibrium dissociation constants (*K*_D_) were determined by fitting the experimental data to a 1:1 Langmuir binding model. ACLYana-1-pTza and ACLYana-3-pTza peptides were used to determine affinity to 1-pHis and 3-pHis Fabs, respectively (Fig. 3 *A*–*E* and Table 1). The ^1^H NMR analysis of ACLYana peptide showed that the peptide undergoes time-dependent phosphorylation in presence of phosphoramidate and that the reaction mixture contains both 1- and 3-pHis peptide isomers (*SI Appendix*, Fig. S7). The two isomers were separated from each other by passing the reaction through affinity columns containing 1-pHis mAbs using SC1-1 or SC50-3 IgGs or 3-pHis mAbs using SC39-4, SC44-8, and SC56-2 IgGs. The eluates thus contained either predominantly 1-pHis or 3-pHis peptides. These peptides were used to determine the affinity to their respective isomer class of pHis Fabs (Fig. 3 *F*–*J*, Table 1, and *SI Appendix*, Fig. S5). SC1-1 and SC50-3 Fabs, displayed an affinity with *K*_D_ value of 25 nM and 16 nM, respectively, with 1-pTza peptide, while their affinity to 1-pHis peptides was about two to four times weaker than the 1-pTza peptides. Whereas SC39-4 and SC56-2 Fabs had an affinity of 67 nM and 97 nM, respectively, for the 3-pTza peptide, the affinities tended to be 10 to 25 times weaker for 3-pHis peptides. In contrast, SC44-8 Fab had a significantly higher affinity (*K*_D_ value of 0.24 nM) toward the 3-pTza peptide, exhibiting a dissociation rate 70 times slower than the other pHis Fabs. This experimental observation is well supported by the higher number of hydrogen bonds that support the SC44-8:3-pTza interaction compared to other pHis Fabs (*SI Appendix*, Table S5). However, the SC44-8 Fab affinity toward the 3-pHis peptide was dramatically reduced by 75-fold compared to the 3-pTza peptide. There are at least three reasons for the lower affinity of the mAbs for pHis peptides compared to pTza peptides: The continuous hydrolysis of phosphate from the pHis peptides during the course of the experiment (*SI Appendix*, Fig. S6), a reduction in the number of hydrogen bond interactions due to one less nitrogen in the imidazole ring compared to triazolyl ring (Fig. 4 and *SI Appendix*, Fig. S8), and inherently different electronic properties of the imidazole and triazolyl rings in His and Tza, respectively (20).

**Table 1.** Interaction kinetics of pHis Fabs with pTza and pHis peptides

Fab type	ACLYana-1/3-pTza peptide (AGAG-1/3-pTza-AGAG) - Cognate type	ACLYana-1/3-pHis peptide (AGAG-1/3-pHis-AGAG)
SC1-1		
*K*_D_, M	2.48 × 10^−8^ ± 1.14 × 10^−10^	9.51 × 10^−8^ ± 5.56 × 10^−10^
* k*_*a*_, 1/Ms	8.13 × 10^4^ ± 3.47 × 10^2^	9.73 × 10^4^ ± 5.51 × 10^2^
* k*_*d*_, 1/s	1.99 × 10^−3^ ± 3.19 × 10^−6^	9.25 × 10^−3^ ± 1.33 × 10^−5^
SC50-3		
*K*_D_, M	1.6 × 10^−8^ ± 6.8 × 10^−11^	2.83 × 10^−8^ ± 1.75 × 10^−10^
* k*_*a*_, 1/Ms	8.51 × 10^4^ ± 3.03 × 10^2^	1.75 × 10^5^ ± 1.01 × 10^3^
* k*_*d*_, 1/s	1.36 × 10^−3^ ± 3.19 × 10^−6^	4.94 × 10^−3^ ± 1.06 × 10^−5^
SC39-4		
*K*_D_, M	6.7 × 10^−8^ ± 4.47 × 10^−10^	7.99 × 10^−7^ ± 3.33 × 10^−8^
* k*_*a*_, 1/Ms	1.64 × 10^5^ ± 1.06 × 10^3^	1.47 × 10^5^ ± 5.78 × 10^3^
* k*_*d*_, 1/s	1.1 × 10^−2^ ± 1.6 × 10^−5^	1.17 × 10^−1^ ± 1.6 × 10^−3^
SC44-8		
*K*_D_, M	2.35 × 10^−10^ ± 4.87 × 10^−12^	1.82 × 10^−8^ ± 7.15 × 10^−11^
* k*_*a*_, 1/Ms	1.56 × 10^5^ ± 4.57 × 10^2^	9.36 × 10^4^ ± 3.19 × 10^2^
* k*_*d*_, 1/s	3.66 × 10^−5^ ± 7.5 × 10^−7^	1.7 × 10^−3^ ± 3.32 × 10^−6^
SC56-2		
*K*_D_, M	9.66 × 10^−8^ ± 1.11 × 10^−9^	2.42 × 10^−6^ ± 7.36 × 10^−8^
* k*_*a*_, 1/Ms	9.73 × 10^4^ ± 1.11 × 10^3^	7.56 × 10^4^ ± 2.17 × 10^3^
* k*_*d*_, 1/s	9.39 × 10^−3^ ± 1.78 × 10^−5^	1.83 × 10^−1^ ± 1.81 × 10^−3^

### Isomer Specificity of pHis Fabs.

The use of nonisomerizable pHis analogs resulted in the development of antibodies that exclusively recognize either 1-pHis or 3-pHis isomers. Analysis with PyIgClassify (28) shows that the canonical conformations of all of the CDRs in 1-pHis Fabs are the same, and that they share common conformations with CDRH2, CDRL1, and CDRL2 in the 3-pHis Fabs (*SI Appendix*, Table S6). These two isomer classes of Fabs differ primarily in the conformations of CDRH3 and CDRL3, which also provide the primary contact points for the triazolyl group. However, there is only 30% sequence identity in the CDR loop residues between the two isomer classes. These differences in the primary and tertiary structure of the CDR loops in 1- and 3-pHis Fabs together determine the shape and chemical complementarity that allows them to differentiate between the two pTza and pHis isomers.

The N1 and N3 positions on the imidazole group of pHis that undergo phosphorylation are equivalent to C5 and N3 on 1-pTza and N2 and C4 on 3-pTza, respectively (Fig. 1*A*). In the case of SC1-1:NM23-1-pTza and SC50-3:NM23-1-pTza, Ser100A of CDRH3 forms hydrogen-bond interactions with the N3 nitrogen on the triazolyl group (equivalent to the N3 position of imidazole). So, this interaction is able to form when the SC1-1 or SC50-3 bind to a pHis peptide and is critical in determining specificity (Fig. 4 *A* and *B*). For SC39-4:ACLYana-3-pTza, Asn91 in CDRL3 makes hydrogen-bond interactions with the N1 nitrogen on the triazolyl group but this interaction is not conserved in the 3-pHis antigen, as it is occupied by a carbon atom (Fig. 4*C*). However, Asn33 in CDRH1 of SC39-4 makes hydrogen bond interactions with the N2 on pTza (equivalent to N1 of imidazole) through a water molecule, which might play role in isomer specificity (*SI Appendix*, Fig. S8*A*). The triazolyl group from the bound-peptide of SC44-8:ACLYana-3-pTza forms hydrogen bonds with Tyr50 in CDRH2, Asn99 in CDRH3 and His91 in CDRL3 of SC44-8. The interaction with Tyr50 is important, as the nitrogen that it interacts with on pTza (N2) is equivalent to the N1 on imidazole and hence Tyr50 is critical for isomer specificity determination (Fig. 4*D*). SC44-8:ACLYana-3-pTza also has a water-mediated interaction with N3 on the triazolyl group through Gly33 in CDRH1, but no equivalent interaction in pHis peptides (*SI Appendix*, Fig. S8*B*). In SC56-2:ACLYana-3-pTza, Tyr50 in CDRH2 and Ser99 in CDRH3 interact with the N1 and N3 nitrogens on the pTza moiety respectively, but imidazole ring lacks a nitrogen at these positions (Fig. 4*E*). SC56-2 also has two water molecules that mediate hydrogen bonding between Gly33 in CDRH1 and Lys94 and Ser99 in CDRH3 with the N3 nitrogen on pTza group, which has no equivalent nitrogen on imidazole (*SI Appendix*, Fig. S8*C*). From these structural snapshots, SC56-2 appears to lack credible hydrogen bonding with the N1 nitrogens on the imidazole ring. However, van der Waals interactions and shape complementarity in the CDR region might be the guiding principles in distinguishing between the isoforms.

To gain further insights into pHis isoform binding specificity, DSF experiments were performed to determine the thermal stability of the antibodies in presence of their noncognate peptides (1-pHis Fabs with 3-pTza peptides and 3-pHis Fabs with 1-pTza peptides). The thermal stability was increased in presence of a cognate peptide (Fig. 2 *F* and *G*) but remained unaffected by presence of a noncognate peptide (*SI Appendix*, Fig. S8 *D* and *E*), therein supporting the selectivity within these interactions. Further, BLI experiments were performed on pHis Fabs in presence of cognate and noncognate peptides to confirm our thermal stability analysis results (Fig. 4*F*), and again there was a minimal signal intensity for association indicating no or weak interactions with noncognate peptides, whereas cognate peptides interacted well with the pHis Fabs.

### pHis Fabs Are Sequence Independent and Dependent.

**Fig. 5.**
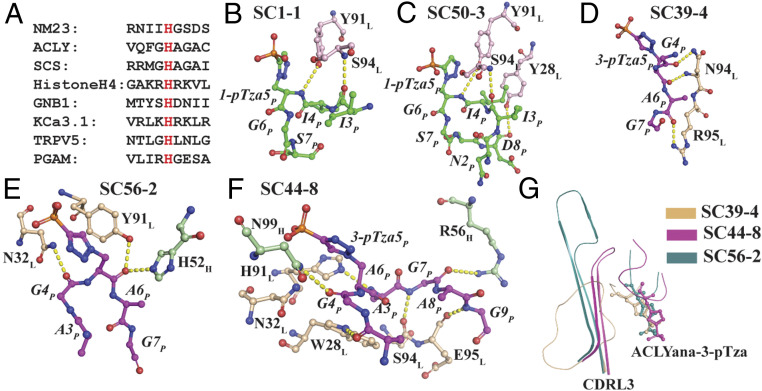
Sequence independence and dependence of pHis Fabs. (*A*) The annotated sites in the proteins that are known to undergo phosphorylation on histidine residues (red font). Interaction map of the peptides (other than phosphate and triazolyl group) with pHis Fabs, SC1-1 (*B*), SC50-3 (*C*), SC39-4 (*D*), and SC56-2 (*E*). These pHis Fabs interact with the peptide backbone rather than side chains, thus conferring the property of sequence independence. SC44-8 (*F*) Fab forms an extensive hydrogen bond network with the GpHAGA motif. (*G*) The CDRL3 loop which makes interaction with the peptide in 3-pHis Fabs is disordered in SC39-4 and far away from peptide in SC56-2, whereas in SC44-8 it is comparatively ordered and proximal to the bound peptide. SC44-8 CDRL3 makes six hydrogen-bonding interactions with the peptide distal to the pTza site, creating steric space constraints that would exclude binding of larger peptide amino acids to the CDR region, thus conferring sequence dependence to GpHAGA motif.

There is no obvious consensus sequence motif among the proteins that undergo phosphorylation on histidine. The sequences surrounding known pHis residues vary significantly (Fig. 5*A*). Inspection of the X-ray structures of our pHis Fabs in complex with 1-pTza and 3-pTza containing peptides reveals that although the antibodies were crystallized with nine-residue peptides, we see ordered electron density for only a few of the peptides’ residues, indicating that the binding pocket is not deep or long enough to accommodate the entire peptide, but only the pHis residue and a few proximal amino acids of the peptide (Fig. 1 *B*–*F*). In the case of SC1-1:NM23-1-pTza, Tyr91 and Ser94 in CDRL3 make hydrogen-bond interactions with amide and carbonyl backbone atoms of two residues on the peptide, respectively (Fig. 5*B*). Along with the above interactions, SC50-3:NM23-1-pTza interacts with C-terminal part of the peptide backbone using Tyr28 from CDRL1 (Fig. 5*C*). In SC39-4:ACLYana-3-pTza, Asn94 and Arg95 in CDRL3 interact with the C-terminal peptide backbone (Fig. 5*D*). In SC56-2:ACLYana-3-pTza, His52 in CDRH2, Asn32 in CDRL1 and Tyr91 in CDRL3 comprise key interactions with the backbone of the 3-pTza peptide (Fig. 5*E*) that also forms one water-mediated hydrogen bond with Asn29 in CDRL1 (*SI Appendix*, Fig. S9*B*). In the SC1-1, SC50-3, SC39-4, and SC56-2 Fab:peptide complexes, two to three hydrogen-bond interactions are made between Fab and peptide, excluding those made with the phosphate and triazolyl moieties (*SI Appendix*, Table S5). This mode of interaction may allow any peptide with pTza/pHis moiety to be recognized by these pHis mAbs conferring sequence independence.

Consistent with its high affinity, SC44-8 displays six hydrogen bonds and multiple van der Waals interactions with the peptide through its light-chain CDR loops. This extensive hydrogen bond network explains why ordered electron density is seen for 6 amino acids in the peptide (GpHAGAG). The N-terminal region of peptide interacts with Trp28, and Asn32 in CDRL1, whereas the C-terminal region is supported by residues in CDRL3 (His91, Ser94, and Glu95) and CDRH2 (Arg56) (Fig. 5*F*). The peptide also binds to Thr52 in CDRH2 through one water molecule (*SI Appendix*, Fig. S9*A*). The proclivity of SC44-8 to bind to the GpHAGAG peptide is endowed by Trp28 in CDRL1 on the N-terminal side and His-91, Ser94, and Glu95 in CDRL3 on the C-terminal side of the peptide. They form a complementary shape and, hence, a steric exclusion region where only small amino acids from the peptide can be accommodated in the binding groove, which is much narrower in SC44-8 (Fig. 5*G*).

Next, we turned to further analyze the peptide selectivity using BLI experiments. Nine amino acid peptide sequences from proteins known to undergo histidine phosphorylation were selected and synthesized (*SI Appendix*, Table S2). The biotinylated peptides ACLY, NM23, SCS, and Common-His were chemically phosphorylated using phosphoramidate (8 h at 23 °C), and affinity-purified using immobilized 1-pHis IgGs or 3-pHis IgGs, as described above. BLI signal intensity was used to assess peptide binding to the Fabs. SC1-1, SC50-3, SC39-4, and SC56-2 had signal intensities in 0.5 to 1 nm for the association (*SI Appendix*, Fig. S10), whereas SC44-8 had maximum signal (2.0 nm) with ACLYana, which has GpHAGAG motif, and a signal of 0.5 nm for ACLY and SCS peptides, which have GpHAGAC and GpHAGAI motifs, respectively. However, there was no signal with NM23 and Common-His peptides, which lack this motif (*SI Appendix*, Fig. S10*D*). This observation suggests that GpHAGA motif is essential for SC44-8 recognition of the pHis substrates. The binding kinetics of the pHis Fabs with this set of peptides indicate that the affinity values are similar for the pHis Fabs SC50-3, SC39-4, and SC56-2, consistent with them binding in a sequence-independent manner. The lack of SC44-8 binding to peptides that lack the GpHAGA motif suggests SC44-8 will be quite selective for pHis phosphosites containing this motif, such as ACLY and SCS (*SI Appendix*, Table S7).

### Lack of Cross-Reactivity with Other Phosphoamino Acids.

The existence of a well-defined cationic phosphate-binding pocket in the pHis antibodies raises the possibility that other phosphoamino acids might be identified by these antibodies. Given the extent and stability of pSer, pThr, and pTyr phosphorylation in the cellular environment, cross-reactivity would be a major drawback for using these mAbs for selective study of His phosphorylation. In earlier studies, polyclonal pHis antibodies were reported to cross-react with pTyr, reducing their utility (21). To investigate if our mAbs cross-react with other phosphoamino acids, we performed DSF and interferometry experiments. Peptides with a “Common” sequence: Gly-Glu-Gln-Leu-X-Asp-Leu-Asn-Ala, where X can be pTyr, pThr, pNLe (phospho-hydroxynorleucine, a phospholysine mimetic), and Lys were synthesized with N-terminal biotin (*SI Appendix*, Table S2). The unphosphorylated Lys peptide was subsequently chemically phosphorylated using phosphoramidate.

**Fig. 6.**
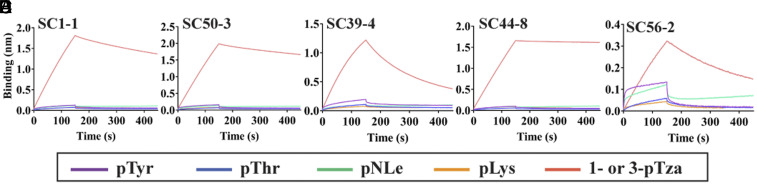
Lack of cross-reactivity with other phosphoamino acid modifications. Traces from the BLI experiments of SC1-1 (*A*), SC50-3 (*B*), SC39-4 (*C*), SC44-8 (*D*), and SC56-2 (*E*) with cognate pTza peptides compared to other phosphoamino acid modifications (pTyr, pThr, pNLe, and pLys). pHis Fabs show no significant binding to pTyr and pThr which have phosphomonoester modifications, pLys, which shares the phosphoramidate bond with the pHis modification, or pNLe, which is a pLys mimetic.

In the DSF experiments, the thermal stability of the pHis antibodies in the presence of pTyr peptide (Common-pTyr) remained unchanged with respect to unliganded antibody, indicating no binding or weak binding (*SI Appendix*, Fig. S11 *A* and *B*). A BLI experiment was carried out to confirm the lack of cross-reactivity. In this experiment, the biotinylated peptides (Common-pTyr, Common-pThr, Common-pNLe, and Common-pLys) were immobilized on streptavidin biosensors and checked for signal intensity for association in presence of pHis antibodies. No association was observed in presence of pTyr or pThr (phosphomonoester modification), or pLys (phosphoramidate linkage as in pHis), or pNLe (phosphomonoester modification and pLys mimic) peptides compared to pTza peptides (Fig. 6). These results establish that this set of pHis mAbs does not cross-react with other phosphoamino acid modifications, and that they exclusively recognize their corresponding pHis isomer. Consistent with this observation, the structural overlay of other phosphoamino acids onto pHis isomers using Flexi-LS-align module in the LS-align program (29) reveals that there would be a spatial limitation or steric exclusion for the other phosphoamino acids, which would prevent their binding to pHis Fabs (*SI Appendix*, Fig. S11 *C*–*E*).

## Discussion

The lack of good biochemical tools has stymied advance in the phosphohistidine field. The recent development of anti-pHis monoclonal antibodies using nonhydrolyzable analogs of pHis has marked a new beginning in the exploration of the role of pHis in mammalian biology (23). These antibodies, most of which are commercially available (*SI Appendix*, *Supplementary Methods and Materials*), have aided in the evolution of our understanding of the pHis modification from being an enzyme intermediate to an essential component in ion channel regulation, tumorigenesis, and so forth (16, 30). pHis mAbs also helped bypass the use of ^32^P autoradiography experiments to study the kinetics of a two-component system (31). Further details of the studies that used pHis mAbs and their significance are tabulated in *SI Appendix*, Table S8. In addition, pHis mAbs have also been used to develop an immunoaffinity purification (IAP) method to enrich pHis peptides from tryptic digests of HeLa cell proteins under nonacidic conditions for analysis by collision-induced dissociation MS/MS. This method uncovered 77 new pHis sites together with previously known pHis sites. Validating these sites will expand on pHis protein candidates in eukaryotes (32).

Given the broad scope and increasing applications of pHis antibodies, we became interested in the mechanism of antigen recognition with the goal of further improving their affinity and specificity against various substrates. Here, we report the crystal structures of Fab fragments of two 1-pHis–selective mAbs (SC1-1 and SC50-3) and three 3-pHis–selective mAbs (SC39-4, SC44-8, and SC56-2) bound to cognate pTza peptides that represent structures of antibodies that target the pHis modification. We provide here structural rationales and experimental validation of the properties of phosphate binding, isomer specificity, sequence dependence/independence, and lack of cross-reactivity to other phosphoamino acids. The five pHis antibodies/Fabs that were developed against the two positional pHis isomers differ from each other in terms of CDR length and sequence resulting in differential properties. Peptide recognition by the pHis Fabs represents the classic paradigm of antibody–peptide interaction. All of these antibodies possess an interface area of 200 to 400 Å^2^ involved in peptide interaction, which is typical for an antibody–peptide interaction (33). The phosphate moiety is recognized through electrostatic interaction with a cationic groove formed by the CDR region, which is supported by five to nine hydrogen bonds (*SI Appendix*, Table S5). The modes of binding of the phosphate moiety in the CDR region of the 1-pHis and 3-pHis mAbs are different, but their features resemble those of two superfamilies of phosphate-binding proteins. The key feature of phosphate binding by the 1-pHis Fabs is the participation of Arg95 in CDRH3 loop in forming salt bridges with the phosphate; this type of phosphate interaction is shared with two phosphate-binding superfamilies, FMN-linked oxidoreductases and PRTase, as well as SH2 and PTB domain-containing proteins that recognize pTyr modifications (34, 35). In contrast, the phosphate-binding site in the 3-pHis Fabs features the presence of a Gly in the phosphate-binding pocket that loosely recapitulates P-loop or Walker A motifs (GXXXX). The Gly makes hydrogen-bond interactions through its main-chain amide groups and also aids the neighboring amino acids in obtaining an optimal conformation for binding with the phosphate group (35). In SC44-8:ACLYana-3pTza, Gly96 (*i*), Gly98 (*i* + 2), and Asn99 (*i* + 3) interact with the phosphate group through main-chain amide groups generating a phosphate-binding motif, called a “nest” that is present in many P-loop–containing proteins (36). This structural convergence of the phosphate-binding motifs suggests that the molecular evolution that occurred in enzymes and the somatic hypermutations that generate antibody CDRs follow similar chemical principles in optimizing phosphate-binding interactions.

The triazolyl group from the phosphotriazolylalanine moiety is not involved in any π–π stacking interactions with CDR residues in the pHis Fabs. However, the direct and water-mediated hydrogen bonds formed with the N3 (N3 equivalent in imidazole ring of His) in 1-pHis Fabs and N2 (N1 equivalent in imidazole ring of His) in 3-pHis Fabs means the topography of the CDR can exclude nonisoform specific substrate binding. Although the number of interactions that the phosphate moiety and triazolyl group make with the antibody outweigh those made by the peptide residues, the cumulative binding-free energy contributed by a few hydrogen-bond interactions and several van der Waals interactions with the peptide backbone play a vital role in defining the sequence dependence or independence properties of the pHis Fabs (*SI Appendix*, Table S5). SC1-1, SC50-3, SC39-4, and SC56-2 exhibit peptide sequence independence by interacting only with one or two residues adjacent to the pTza residue via the peptide backbone and exposing the side chains and other residues in the peptide to solvent. For various applications involving detection of pHis signals, we recommend the use of either SC1-1 or SC50-3 or a combination of both to study 1-pHis modification, while SC39-4 and SC56-2 offer the best sequence independence in the case of 3-pHis antibodies, although they have lower affinity in the submicromolar range due to faster rates of dissociation.

In contrast, SC44-8 should be used with caution, as it has preference toward binding the GpHAGA motif, which serves as a phosphoenyzme intermediate in enzymes, like ACLY and SCS. This sequence dependency is endowed upon SC44-8 by CDRL3, which is conformationally very stable compared to CDRL3 in SC39-4 and situated more proximal to the CDR groove compared to CDRL3 in SC56-2; this results in the Ala-Gly-Ala residues on the C-terminal side of pHis being selected because of a spatial constraint that sterically excludes bulkier amino acids (Fig. 5*G*). This finding does not come as a surprise, because the GpHAGA motif will have been present among the peptides in the degenerate 3pTza peptide library that was used for immunization (23). However, to study the total cellular pHis proteome, we prefer to use a combination of all five pHis mAbs to exploit the best of the properties of each antibody (32).

Our DSF and BLI analyses establish that the pHis mAbs do not cross-react with other phosphoamino acid modifications, such as pTyr, pThr, pNLe, and pLys. The three-dimensional overlay of phosphoamino acids on pHis also hints at the spatial and steric differences in the functional groups that might contribute to their noncross-reactivity (*SI Appendix*, Fig. S11). The measured affinities of pHis Fabs with pTza peptides are 2 to 75 times higher than for pHis peptides, largely because of the nonhydrolyzable nature of the pTza as opposed to pHis, and the differences in atoms, shape, and electronics of pTza versus pHis moieties (20) (Fig. 3 and Table 1). While 1- and 3-pHis peptides undergo time-dependent dephosphorylation, pHis mAbs stabilize these peptides by sequestering the phosphoramidate linkage of the pHis peptide from the solvent and thus decreasing the rate of dephosphorylation (*SI Appendix*, Fig. S6). This observation has implications for the retention of the sensitive pHis modification during experimental procedures that use these antibodies.

Although phospho-specific antibodies are widely used in research, very few structures are available, with the first being reported in 2012 for a chicken mAb against tau peptide with a pThr modification (37). James Wells’ group determined structures of engineered pSer, pThr, and pTyr antibodies developed from a single nest motif-specific mAb scaffold (38). They also solved structures of the 4G10 anti-pTyr mAb and its engineered counterpart (39). As the phosphate moiety is the common feature among all these phosphoamino acid modifications, we checked if these antibodies share their CDR regions or antigen recognition patterns. In case of the antiphospho-tau antibody and the engineered pSer, pThr, and pTyr antibodies, their binding pattern involves use of a nest scaffold in the CDRH2 loop to make direct hydrogen-bond interactions with the phosphate. A similar pattern of recognition is also observed in SC44-8, but the nest is located on the CDRH3 loop region instead (Fig. 2*D* and *SI Appendix*, Fig. S1).The engineered 4G10 antibody and 4G10 have a different mode of phosphate binding compared to the aforementioned antibodies, but their binding is similar to our 1-pHis antibodies. These antibodies make salt bridge interactions with the phosphate through Arg residues in CDRH3. Although the pattern of recognition of phosphate is common among these antibodies, the CDR regions do not share much sequence identity and the buried surface area of phosphate is different. These two factors play an important role in determining the depth of the CDR binding pocket, which further distinguishes the phosphoamino acid specificity. Combining the information from different phosphoamino acid-specific antibodies may facilitate future engineering to make the antibodies more mutually exclusive in terms of antigen recognition and further minimize cross-talk.

In addition to exploiting these antibodies to study the pHis proteome, the structural data are being used to guide antibody-engineering approaches, such as rational design to improve the affinity and specificity of pHis mAbs and directed evolution to make pHis sequence-specific antibodies. Improved antibodies together with optimized techniques (40–42) to study the pHis modification in in vitro and in vivo conditions will be invaluable for studies of the intracellular localization, endogenous pHis kinetics, and uncovering more histidine kinases and phosphatases and their binding partners.

Overall, our studies provide insight into the structural aspects of the pHis Fabs and their differential recognition of pHis proteins. They provide a guide for choosing which pHis mAbs are most useful for pHis research and a framework for structure-guided antibody engineering of powerful second-generation antibodies. While these pHis antibodies continue to fuel studies of the labile posttranslational modifications, their success should inspire the development of antibodies against other labile phosphoamino acid modifications, thus expanding the biochemical toolbox for studying posttranslational modifications.

## Materials and Methods

### Conversion of Hybridoma Clones into Recombinant Clones.

pHis rabbit hybridoma cells were grown in medium containing 1× HAT 240E, RPMI-1640 and 10% FBS, and mRNA was isolated from 10^6^ cells using the NucleoSpin RNA isolation kit (Machery-Nagel). The mRNA was converted into cDNA using the polyT primer in the SuperScript III First-Strand synthesis kit (Invitrogen). The variable chains from the cDNA were amplified using a set of primers as described by Rader (43). The PCR products were cloned into pFUSEss-CHIg rG and pFUSE2ss-CLIg rk1 vectors (Invivogen) for the heavy chain and light chain, respectively. The positive clones were confirmed by sequencing. For Fab expression, a 6× Histidine tag and stop codon were inserted after Kabat position 215 in the heavy chain. The light chain of Fab SC50-3 was not successfully cloned, but as its sequence is very similar (93% identical in the CDR sequence) to that of Fab SC1-1, the Fab was expressed with the SC50-3 heavy chain and SC1-1 light chain. All other Fab pairs had unique light- and heavy-chain sequences.

### Transfection, Expression, and Purification of the IgGs and Fabs.

The plasmids for heavy and light chains for IgGs and Fabs were isolated using Qiagen maxi prep kits and cotransfected into Expi293F cells using the Expi293F Transfection kit (ThermoFisher Scientific) following the supplier’s protocol. Expi293F cells were grown in Expi293F medium at 37 °C, 8% CO_2_ with 125 rpm shaking. For transfection, the Expi293F cells were seeded at a density of 1 to 2 × 10^6^ cells/mL in 200 mL of Expi293F medium. On the day of transfection, DNA solution was prepared by mixing 135 μg of heavy-chain DNA vector with 65 μg of light-chain DNA vector in 10 mL of Opti-MEM reduced serum medium. Expifectamine 293 solution was prepared by mixing 540 μL of Expifectamine 293 with 10 mL of Opti-MEM and incubated for 5 min. The lipid–DNA complexes were prepared by adding DNA solution to the Expifectamine 293 solution, gently swirling, and incubating at 23 °C for 20 min before adding to the culture. After incubation for 18 h, cells were fed with 1 mL Expifectamine 293 Enhancer 1 and 10 mL of Expifectamine 293 Enhancer 2. After a total of 5 d of incubation, the cell culture was sterile filtered and cell supernatant was incubated with GE Excel Ni Sepharose beads (GE Healthcare) for 1 h at 4 °C on a Nutator. The His-tagged Fab protein was eluted by step-gradient elution using imidazole in 20 mM Tris pH 8.0 and 500 mM NaCl. For pHis IgG proteins, the sterile-filtered cell supernatant was added to the protein-A agarose beads and incubated for 1 h at 4 °C. The pHis IgGs were eluted by 100 mM glycine pH 3.0 solution and the eluate was immediately neutralized by 1 M Tris pH 9.0. The homogeneity of the proteins was checked on an SDS/PAGE gel and the proteins were concentrated using 10-kDa cutoff Millipore centrifugal concentrators. The proteins were further purified on a Sepharose S200 16/60 column (GE Healthcare) equilibrated with 20 mM Hepes pH 8.0 and 150 mM NaCl. The yields of the purified pHis Fabs were 1 to 2 mg and IgGs were 3 to 4 mg from a 200-mL culture.

### Crystallization and Structure Solution.

SC1-1 and SC50-3 proteins were preincubated with NM23-1-pTza peptide (RNII-1-pTza-GSDS) and SC39-4, SC44-8, and SC56-2 were preincubated with ACLYana-3-pTza peptide (AGAG-3-pTza-AGAG) at a molar ratio of 1:5 (Fab:peptide). Protein was screened for crystallization using our Rigaku CrystalMation system, with JCSG Screens 1–4 at 4 °C and 20 °C. The crystallization conditions and corresponding cryoprotectants used are tabulated in *SI Appendix*, Table S9. Data were collected at Advanced Photon Source beamline 23-ID-D, Stanford Synchrotron Radiation Lightsource beamline 12-2, and Advanced Light Source beamline 5.0.3 at 100 K. The diffraction images were indexed, integrated and scaled using HKL-2000 (44). The rabbit antihypusine antibody-FabHpu98.61 (PDB ID code 5DTF) (45) was used as search model for molecular replacement with PHASER (46) to obtain initial phase information. Refinement was carried out by alternating rounds of Refmac5 (47, 48) and model-building software Coot (49). The final models were validated using MolProbity (50) and figures were prepared using PyMOL (Schrodinger) and University of California, San Francisco Chimera (51). Buried molecular surfaces were analyzed with the MS program (52) using a 1.7-Å probe radius. The data collection, refinement statistics, and the model-validation values are reported in *SI Appendix*, Table S1.

### Differential Scanning Fluorimetry.

pHis Fabs (3 µM) were combined with 5× SYPRO Orange protein gel stain (Millipore Sigma) in the presence and absence of 1 mM peptides (pTza or other phosphoamino acid peptides) in buffer containing 50 mM Hepes pH 8.0 and 150 mM NaCl. The mixture was transferred into a 96-well plate and subjected to heating at a ramp rate of 0.03 °C/s on QuantStudio3 (Applied Biosystems) with ROX reporter and x1-m3 filter from 25 °C to 95 °C. The data collected using the corresponding software were fitted to the two-state transition curve and the inflection point (*T*_m_) was measured using Boltzmann equation in GraphPad Prism software. Each experiment was performed three times.

### Biolayer Interferometry.

The binding kinetics of pHis Fabs with the peptides (pTza and pHis) were evaluated with an Octet RED96e (FortéBio). Streptavidin-coated biosensors (FortéBio) were presoaked in buffer containing 50 mM Hepes pH 8.0, 150 mM NaCl, 0.05% Tween-20, and 0.2% BSA for 15 min. The biotinylated pTza peptides or pHis peptides (20 nM) with and without phosphoramidate treatment were coated onto these streptavidin biosensors and analyzed for their binding to varying concentrations of the pHis Fabs with an association time of 120 s and dissociation time of 200 s. The entire set of experiments was repeated three times. The data were analyzed using the Octet software (FortéBio) and the rates of association and dissociation were obtained by global fitting data to the 1:1 Langmuir interaction model.

## Supplementary Material

Supplementary File

## Data Availability

The X-ray coordinates and structure factors of the pHis Fab:peptide complexes have been deposited in the Protein Data Bank, https://www.rcsb.org/ (PDB ID codes 6X1S [SC1-1:NM23-1-pTza], 6X1T [SC50-3:NM23-1-pTza], 6X1U [SC39-4:ACLYana-3-pTza], 6X1V [SC44-8:ACLYana-3-pTza], 6X1W [SC56-2:ACLYana-3-pTza]). All other study data are included in the article and supporting information.
