# Supplementary material for: Structural basis for differential recognition of phosphohistidine-containing peptides by 1-pHis and 3-pHis monoclonal antibodies
Source: Proc Natl Acad Sci U S A. 2021 Feb 5;118(6):e2010644118. doi: 10.1073/pnas.2010644118 (PMC8017925; doi:10.1073/pnas.2010644118)
Supplement: Supplementary File [file pnas.2010644118.sapp.pdf]

PNAS
www.pnas.org

Supplementary Information for

Structural basis for differential recognition of phosphohistidine-containing peptides by 1-pHis and 3-pHis monoclonal antibodies

Rajasree Kalagiri[a], Robyn L. Stanfield[b], Jill Meisenhelder[a], James J. La Clair[c,d], Stephen R. Fuhs[a], Ian A. Wilson[b,e], Tony Hunter[a,*]

[a]Molecular and Cell Biology Laboratory, Salk Institute for Biological Studies, La Jolla, CA 92037, United States.

[b]Department of Integrative Structural and Computational Biology, The Scripps Research Institute, La Jolla, CA 92037, United States.

[c]Department of Chemistry and Biochemistry, University of California, San Diego, La Jolla, CA 92093, United States.

[d]Jack H. Skirball Center for Chemical Biology and Proteomics, Salk Institute for Biological Studies, La Jolla, CA 92037, United States.

[e]The Skaggs Institute for Chemical Biology, The Scripps Research Institute, La Jolla, CA 92037, United States.

*Correspondence should be addressed to T.H.
Email: hunter@salk.edu

This PDF file includes:

SI methods and materials
Figures S1 to S11
Tables S1 to S9
SI References

Supplementary appendix

Supplementary methods and materials

Peptide Synthesis. Peptides used for the crystallization were synthesized at Sanofi as described in Fuhs et al. (1). Peptides used in the DSF and BLI experiments were synthesized by the Salk Peptide Synthesis Core. These peptides were N-terminally biotinylated and C-terminally amidated. They were synthesized using standard Fmoc chemistry on a Gyros Protein Technologies Tribute peptide synthesizer with UV monitoring. Following the deprotection, peptides were analyzed by mass spectrometry and analytical HPLC by the Proteomics Core at Salk. The peptides were HPLC purified to 99% purity by 21st Century Biochemicals, Inc. The Fmoc-pTza's were synthesized at 10 g scale for incorporation into the peptides.

Phosphoramidation reaction. The small molecule phosphate donor phosphoramidate was used to phosphorylate the peptides containing His (Table S2). Phosphoramidate was synthesized based on Wei and Matthew's method (2) and stored in a desiccator. 20 nanomoles of peptides were added to one micromole of the phosphoramidate in 50 mM Hepes pH 8.0 and 150 mM NaCl buffer. This mixture was incubated for 8 h at 23 °C.

Immunoaffinity purification of pHis peptides. Individual immunoaffinity columns were prepared for the two pHis isoforms. 125 μg of 1-pHis IgGs (equal mixture of SC1-1 and SC50-3) and 125 μg of 3-pHis IgGs (equal mixture of SC39-4, SC44-8 and SC56-2) were added separately to 500 μL of Protein A agarose beads in the presence of 5 mM BS^3 crosslinker and incubated for 45 min on the Nutator. Excess crosslinker was removed and columns were washed thoroughly using 50 mM Hepes pH 8.0 with 150 mM NaCl. Peptides from the overnight phosphoramidation reaction were added to the affinity column containing either 1-pHis IgGs or 3-pHis IgGs. The incubation was continued for one hour at RT. The supernatant was removed, and the beads were washed thrice with the Hepes buffer. Each phosphorylated peptide was eluted from the corresponding isomer affinity column using 600 μL of 2 M imidazole and the eluate was immediately used for the Biolayer interferometry assays.

^{1}H NMR of ACLYana peptide. A mixture of 5 mM ACLYana peptide and 250 mM phosphoramidate in 20 mM d_{18}-Hepes, pH 8.0, 50 mM NaCl in D_2O was incubated at 23 °C and monitored over the course of 29 h. Each time point collected from a 50 μL volume of which 35 μL was transferred to a 1.7 mm NMR capillary tube and ^{1}H NMR data were acquired on Bruker Advance III 600 equipped with a 1.7 mm cryoprobe. Chemical shifts (ppm) were referenced using

the corresponding solvent signals (δ_H 4.79 for D_2O). Delay time (d1) was set to 5 to ensure accurate integrations. The NMR spectra were processed using Mestrenova (Mnova 11.0 Mestrelab Research).

pHis IgG commercial antibodies. Some of the pHis IgG antibodies are commercially available from EMD Millipore. Anti-N1-Phosphohistidine (1-pHis) antibody- SC1-1 (MABS1330), Anti-N1-Phosphohistidine (1-pHis) antibody- SC50-3 (MABS1341), Anti-N3-Phosphohistidine (3-pHis) antibody- SC39-6 (MABS1351) and, Anti-N3-Phosphohistidine (3-pHis) antibody- SC56-2 (MABS1352).

Supplementary figures

Fig. S1.

		CDR1	CDR2	CDR3
SC1-1	VH	31-GHGVI-35	50-SAGAYGRIYYASWAKS-65	95-RSDVGTSVGFDS-102
SC50-3	VH	31-GYGVI-35	50-SAGAYGRIYYPNWARS-65	95-RTNVSTSVGFDS-102
SC1-1	VL	24-QASESVYSNNRLS-34	50-LVSTLAS-56	89-VGYKSSTTDGLA-97
SC50-3	VL	24-QASETVY-NNRLS-34	50-LVSTLAS-56	89-VGYKSSTTDGLA-97
SC39-4	VH	31-RYNMG-35	50-WIPFRGSLKYATWATG-65	95-SSDGF--DL-102
SC44-8	VH	31-SYGFS-35	50-YLTAGGRAFYASWAKS-65	95-LGSGNPVAI-102
SC56-2	VH	31-SYGFS-35	50-YLHANGRAYYATWAKS-65	95-IGSVSDVAI-102
SC39-4	VL	24-QASR--DTGDGLI-34	50-KASTVAS-56	89-HSNFYNRWTYG------NA-97
SC44-8	VL	24-QSSQSVWRNKNLA-34	50-AIATLDS-56	89-VGHYGSENDAY------YA-97
SC56-2	VL	24-QSSESIYNNKNLA-34	50-SISTLAS-56	89-VGYYYSGGYYYSGSAAYYA-97

Fig. S1. Depiction of pHis mAbs CDR sequence variability and physical contacts with pTza peptide. The sequence of the CDR regions of heavy and light chains of pHis mAbs. The sequence variability is high among the 3-pHis mAbs (SC39-4, SC44-8 and SC56-2) compared to the 1-pHis mAbs (SC1-1 and SC50-3). The grey shaded residues and grey shade with boxed residues make direct and water-mediated interactions with the phosphate moiety respectively, the dark beige shaded and asterisk signed residues interact with the triazolyl moiety, and the pink shaded and pound signed residues interact with the peptide. The 'nest' motif in CDRH3 region of SC44-8 is boxed. Most of the interactions are through hydrogen bonds, which are listed in SI Appendix, Table S5. The hydrogen bonds are measured using the HBPLUS program (3).

Fig. S2.

(A)

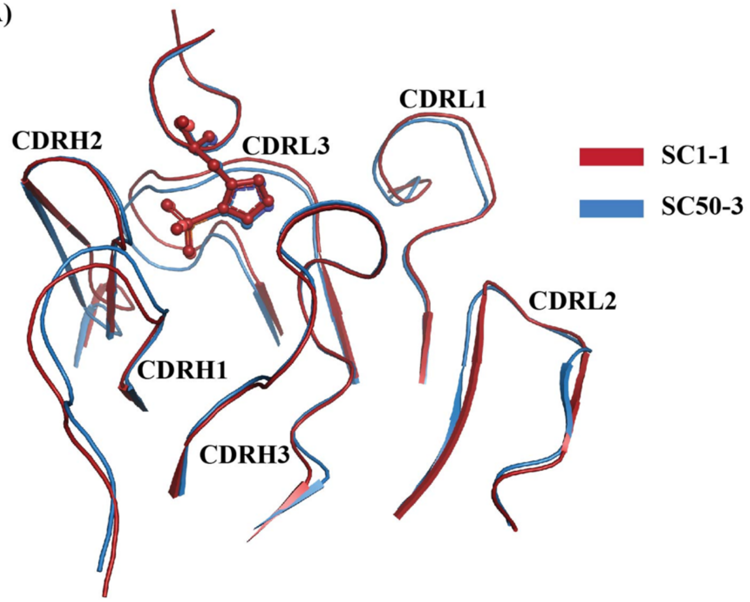

(B)

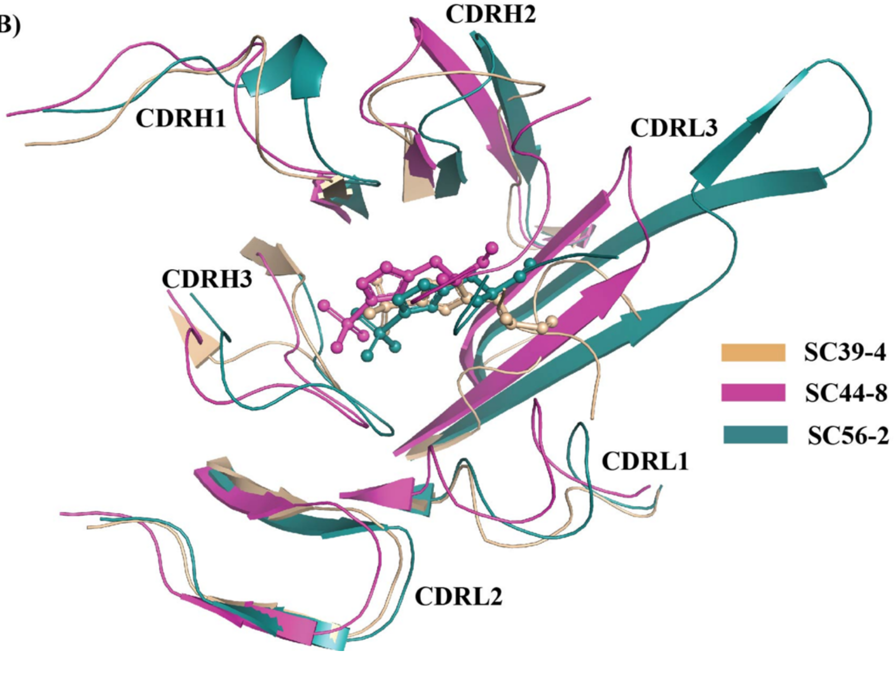

Fig. S2. Structural superposition of pHis Fabs on the Cα carbon. *(A)* Overlay of six CDRs of 1-pHis Fabs, SC1-1 and SC50-3, on their Cα carbon backbone. The RMSDs of CDRs and the bound peptide are very low. *(B)* Overlay of six CDRs of 3-pHis Fabs (SC39-4, SC44-8 and SC56-2) shows higher RMSDs. The high CDR variability in CDRL3 is reflected in the high RMSDs in 3-pHis Fabs.

Fig. S3.

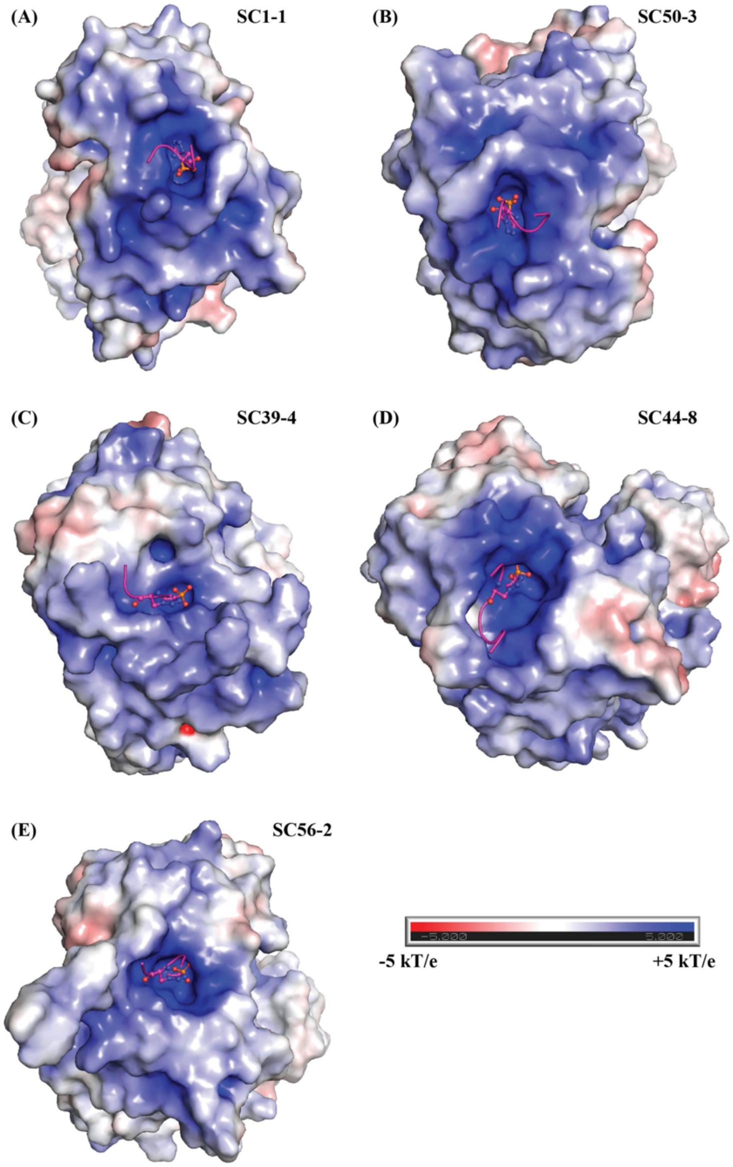

Fig. S3. Electrostatic surface representation of pHis Fabs. The electrostatic potential on the surface of the pHis Fabs, SC1-1 *(A)*, SC50-3 *(B)*, SC39-4 *(C)*, SC44-8 *(D)* and SC56-2 *(E)*, bound to cognate peptides (represented pink) indicate that a strongly positive electrostatic potential of the binding site facilitates the recognition of the phosphate moiety. This surface representation of the pHis Fabs was generated using the Adaptive Poisson-Boltzmann Solver (APBS) in PyMol. The red- and blue-colored regions show the regions with negative (red) and positive (blue) electrostatic potential contoured from −5 to +5 kT/e, respectively.

Fig. S4.

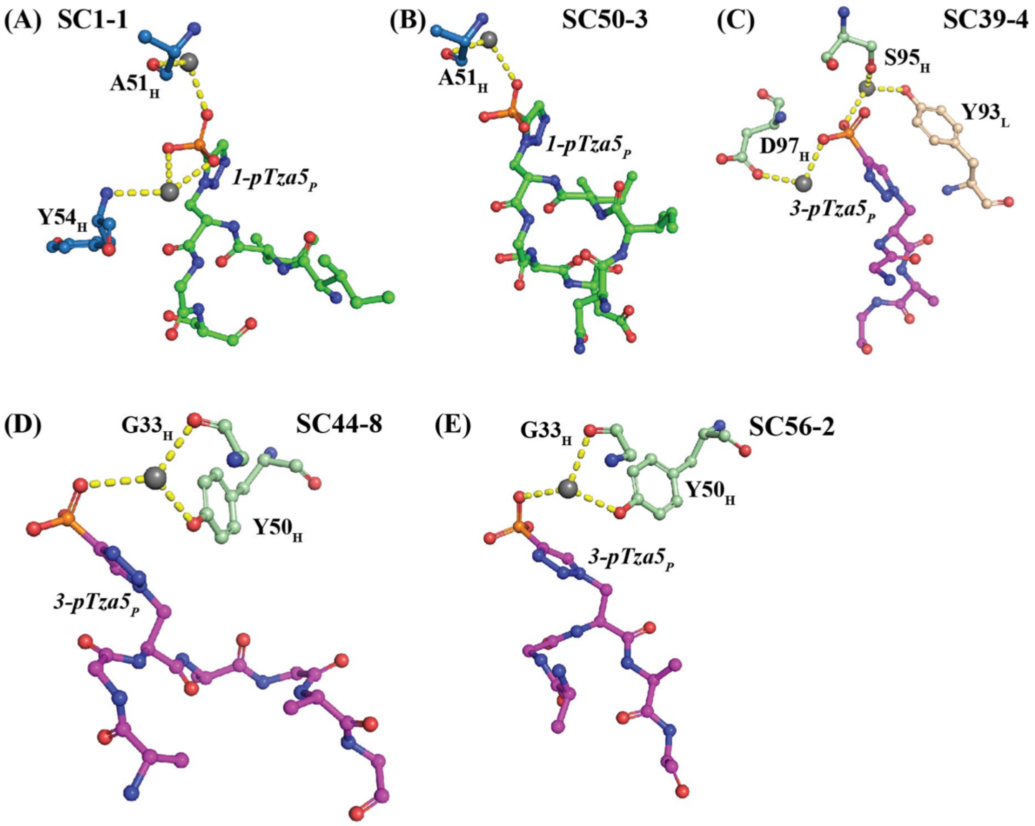

Fig. S4. Water-mediated interactions of the phosphate moiety of the peptide with pHis Fabs. *(A)* Ala51 and Tyr54 in CDRH2 of SC1-1 are involved in hydrogen bonding with the phosphate in the peptide through two water molecules (represented in grey). *(B)* SC50-3 makes only one water-mediated interaction with the phosphate moiety through Ala51. The water which mediates interaction between Ala51 and the phosphate is conserved in 1-pHis Fabs. *(C)* Ser95 and Asp97 in CDRH3 and Tyr93 in CDRL3 make three interactions through two water molecules. *(D)* SC44-8 and *(E)* SC56-2 makes two interactions with Gly33 in CDRH1 and Tyr50 in CDRH2 through one water molecule which is conserved among these two Fabs.

Fig. S5.

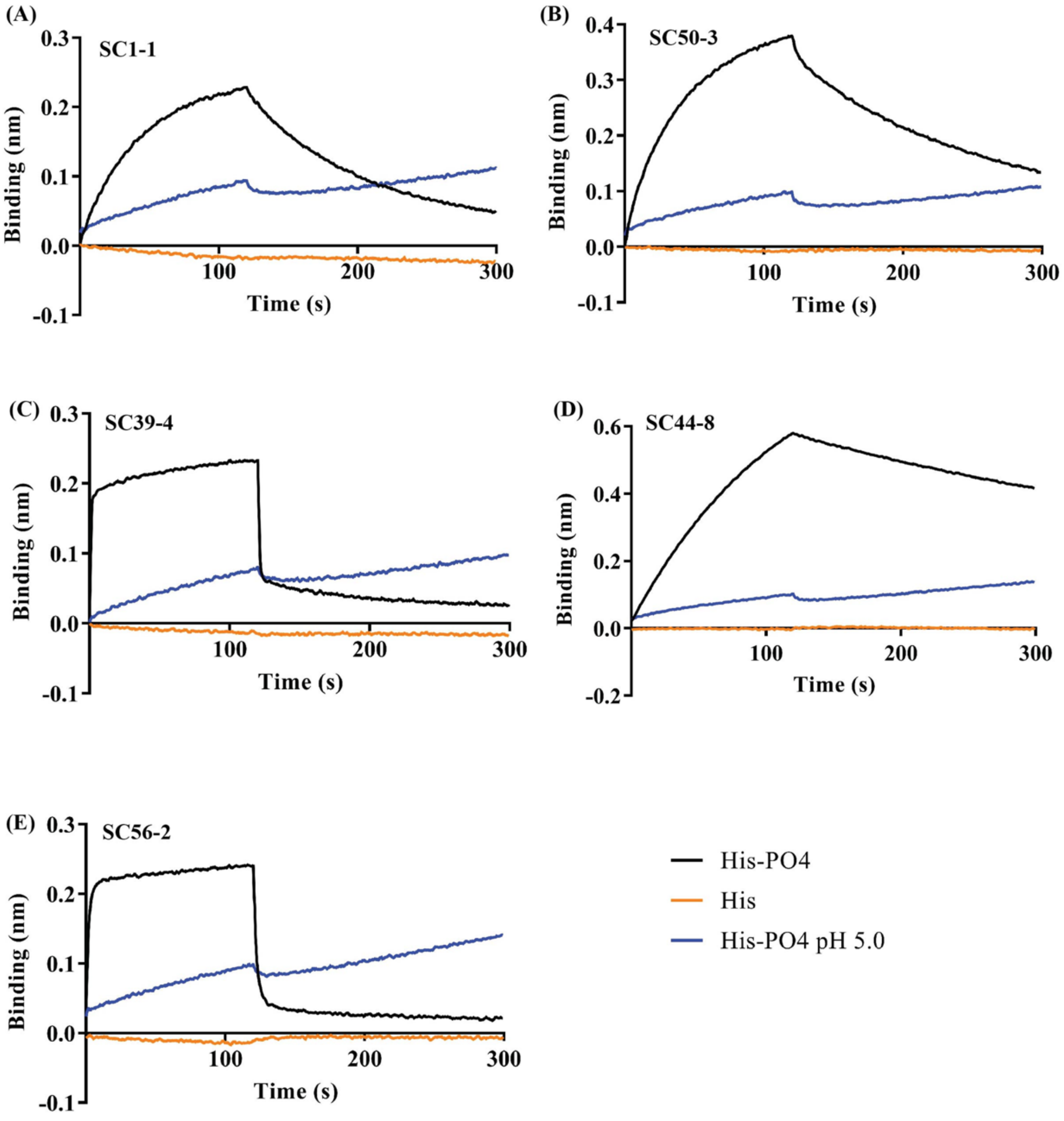

Fig. S5. pHis *versus* His discrimination by pHis Fabs. The BLI plots represent the interaction of pHis Fabs, SC1-1 *(A)*, SC50-3 *(B)*, SC39-4 *(C)*, SC44-8 *(D)*, and SC56-2 *(E)*, with pHis peptides over non-phosphorylated histidine peptides. Binding of pHis Fabs is lost with pHis peptides at acidic pH (pH 5.0).

Fig. S6.

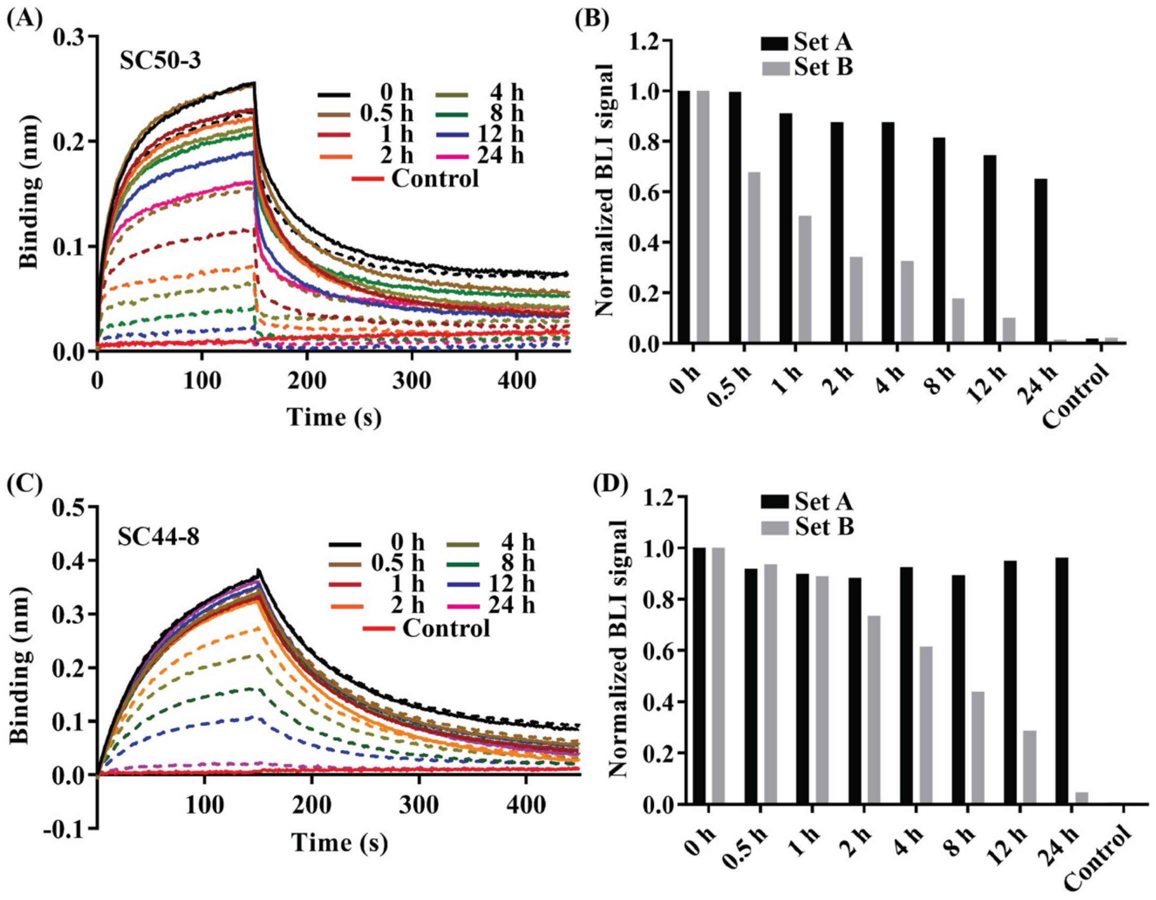

Fig. S6. Stability of 1- and 3-pHis peptides in the presence of pHis mAbs. In the BLI experiment, analytes were SC50-3 *(A and B)* and SC44-8 *(C and D)* Fabs and ligand was the ACLYana peptide. Prior to the experiment, the peptide was phosphorylated with phosphoramidate and divided into two sets - set A and set B. The set A peptide was incubated with 1- and 3-pHis mAbs separately for extended periods of time (0 - 24 h) and set B was not incubated with pHis mAbs. The ACLYana-pHis peptide incubated with the mixture of 1-pHis IgGs (*A*, continuous lines) and 3-pHis IgGs (*C*, continuous lines) has the higher signal intensity at all time points compared to ACLYana-pHis peptide that did not undergo extended periods of incubation with pHis IgGs (discontinuous lines). The BLI signal intensity at the end of association phase was used to compare the rate of hydrolysis with (set A) and without incubation with pHis mAbs (set B). The signal intensities were normalized to the 0 h *(B and D)*. The unphosphorylated peptide was used as a negative control. The data indicate that the pHis mAbs stabilize the pHis peptides by decreasing the rate of dephosphorylation. In the absence of pHis mAb incubation, 1-pHis peptide hydrolyzed faster than 3-pHis peptide.

Fig. S7.

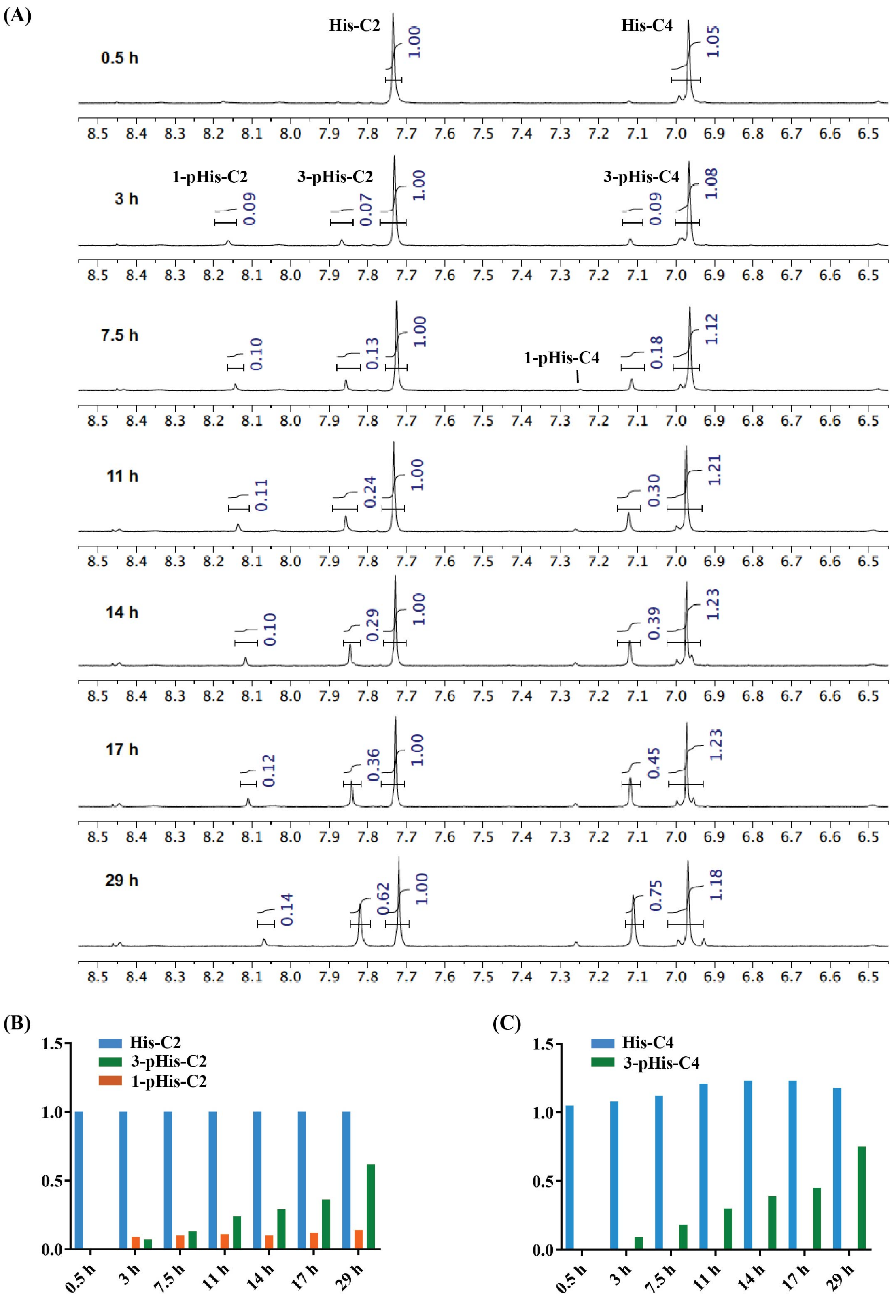
(A)
0.5 h
His-C2
His-C4
1.00
1.05
3 h
1-pHis-C2
3-pHis-C2
3-pHis-C4
0.09
0.07
1.00
0.09
1.08
7.5 h
0.10
0.13
1.00
1-pHis-C4
0.18
1.12
11 h
0.11
0.24
1.00
0.30
1.21
14 h
0.10
0.29
1.00
0.39
1.23
17 h
0.12
0.36
1.00
0.45
1.23
29 h
0.14
0.62
1.00
0.75
1.18
8.5 8.4 8.3 8.2 8.1 8.0 7.9 7.8 7.7 7.6 7.5 7.4 7.3 7.2 7.1 7.0 6.9 6.8 6.7 6.6 6.5
(B)
His-C2
3-pHis-C2
1-pHis-C2
1.5
1.0
0.5
0.0
0.5 h
3 h
7.5 h
11 h
14 h
17 h
29 h
(C)
His-C4
3-pHis-C4
1.5
1.0
0.5
0.0
0.5 h
3 h
7.5 h
11 h
14 h
17 h
29 h

Fig. S7. ^{1}H-NMR time course analysis of phosphorylation of ACLYana peptide. *(A)* ^{1}H-NMR spectra collected at designated time points. Over the course of the reaction, the proton at C2 observed at 7.7 ppm shifts to 7.85 ppm and 8.15 ppm upon phosphorylation at the respective 3-pHis and 1-pHis positions. Likewise, the proton at C4 also shifted from 6.95 ppm to 7.25 ppm and 7.10 ppm when undergoing phosphorylation at 1-pHis and 3-pHis, respectively. There was no observable formation of 1,3-diphosphohistidine. The integrated peaks of the C2 *(B)* and C4 *(C)* imidazole ring protons peaks demonstrate time-dependent formation of ACLYana-1-pHis and ACLYana-3-pHis.

Fig. S8.

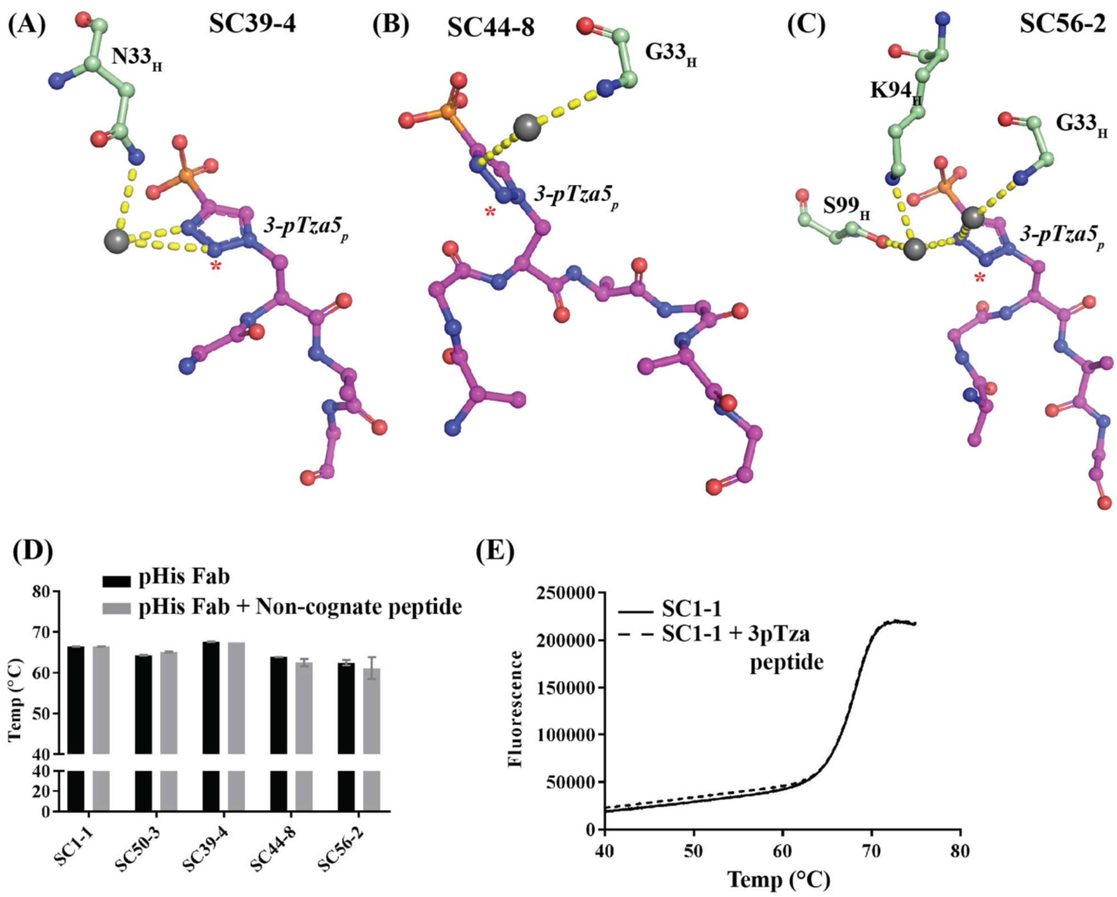

Fig. S8. Isomer specificity of pHis Fabs. 1-pHis Fabs do not make any water-mediated interactions with triazolyl group. *(A)* Asn33 in CDRH1 of SC39-4 makes two interactions with triazolyl group through one water molecule (represented in grey). *(B)* SC44-8 forms hydrogen bonds between Gly33 in CDRH1 to triazolyl group via one water molecule. *(C)* Along with Gly33 in CDRH1, Lys94 and Ser99 in CDRH3 from SC56-2 forms interactions with the triazolyl group through water molecules. The atoms corresponding to nitrogens on the imidazole, which are free to undergo hydrogen bonding, are starred. These interactions may form when pHis mAbs bind to pHis peptides. *(D)* The T_m derived from the DSF analysis of pHis Fabs in presence of their non-cognate pTza peptides shows that there is no change in thermal melt temperature indicating minimum to no cross reactivity across isoform-specific antibodies. *(E)* A representative thermal melt plot of SC1-1 in presence and absence of ACLYana-3-pTza peptide (noncognate).

Fig. S9

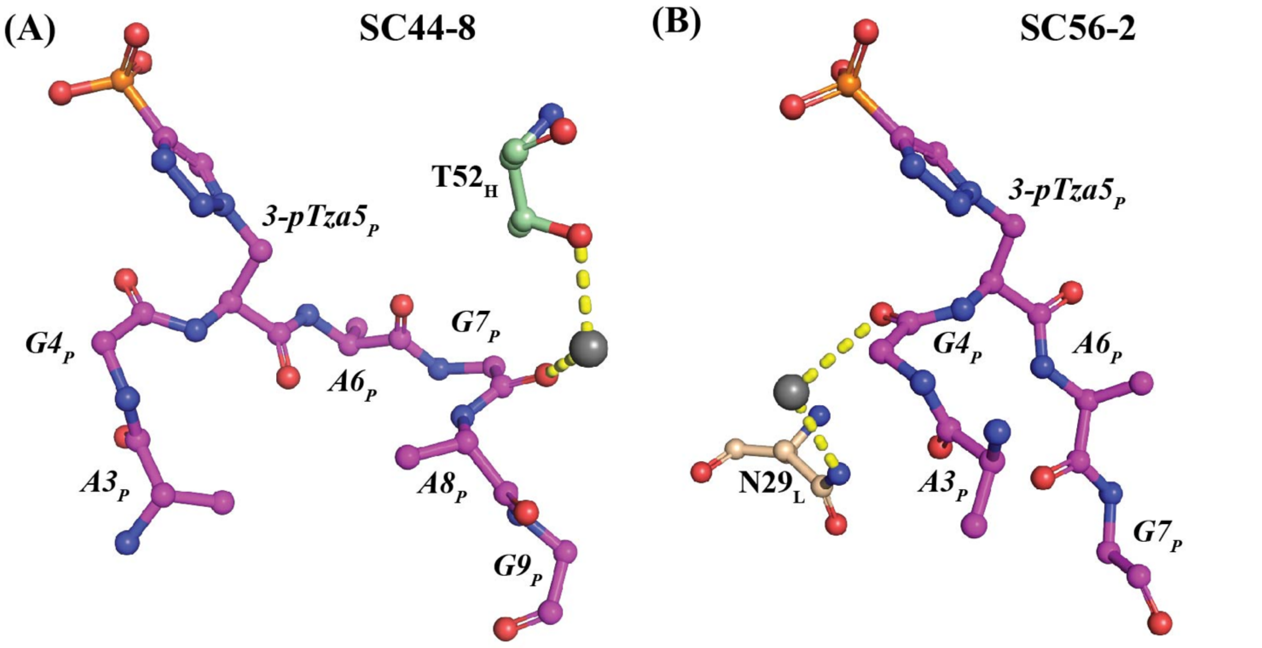

Fig. S9. Water-mediated interactions of peptides with pHis Fabs. *(A)* SC44-8 makes one water-mediated interaction (represented in grey) with the peptide backbone through Thr52 in CDRH2. *(B)* SC56-2 also makes one water-mediated interaction between Asn29 in CDRL1 with the peptide backbone. SC1-1, SC50-3 and SC39-4 do not form any water-mediated interactions as there are no ordered water molecules in the CDR region and most of the peptide is solvent exposed.

Fig. S10.

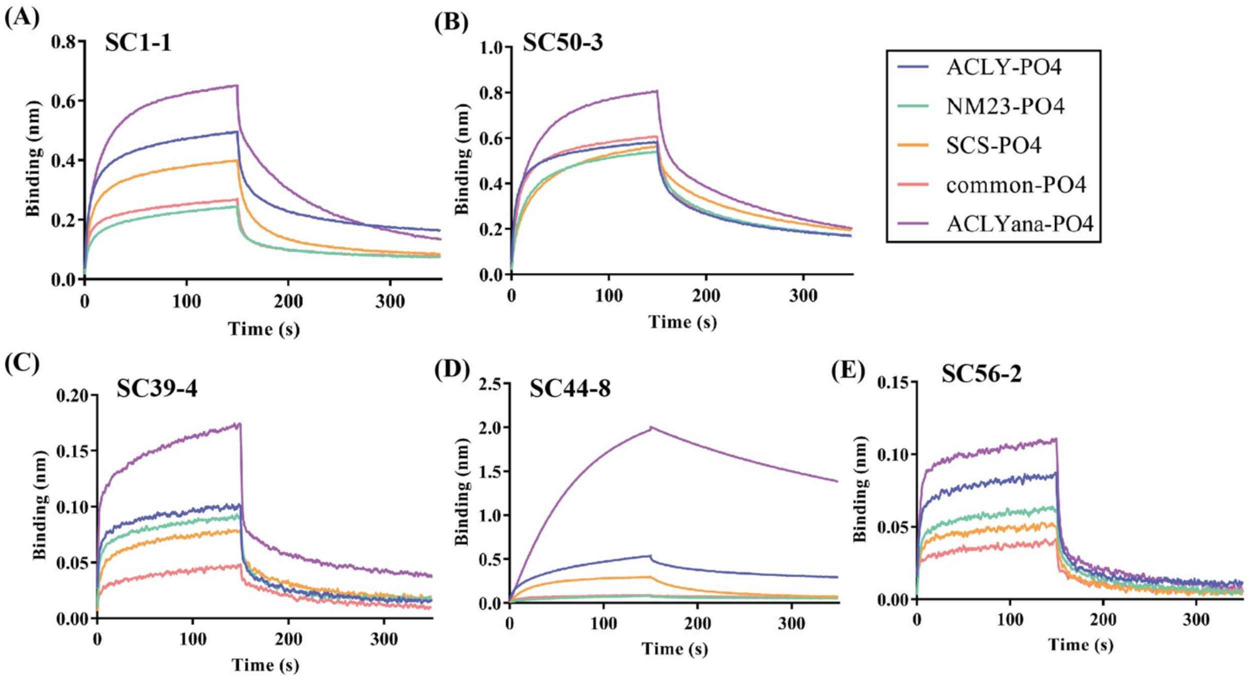

Fig. S10. Sequence independence property of pHis Fabs. BLI studies on SC1-1 *(A)*, SC50-3 *(B)*, SC39-4 *(C)* and SC56-2 *(E)* in the presence of different peptides shows that there is a minimal signal intensity difference with different peptides indicating that these Fabs can bind any of the peptides tested with a pHis modification. SC44-8 *(D)* shows a maximum signal intensity with peptides with a GpHAGA motif (ACLYana, ACLY and SCS) compared to other peptide sequences (NM23 and Common-His peptide). The corresponding affinity values are tabulated in Table S7.

Fig. S11.

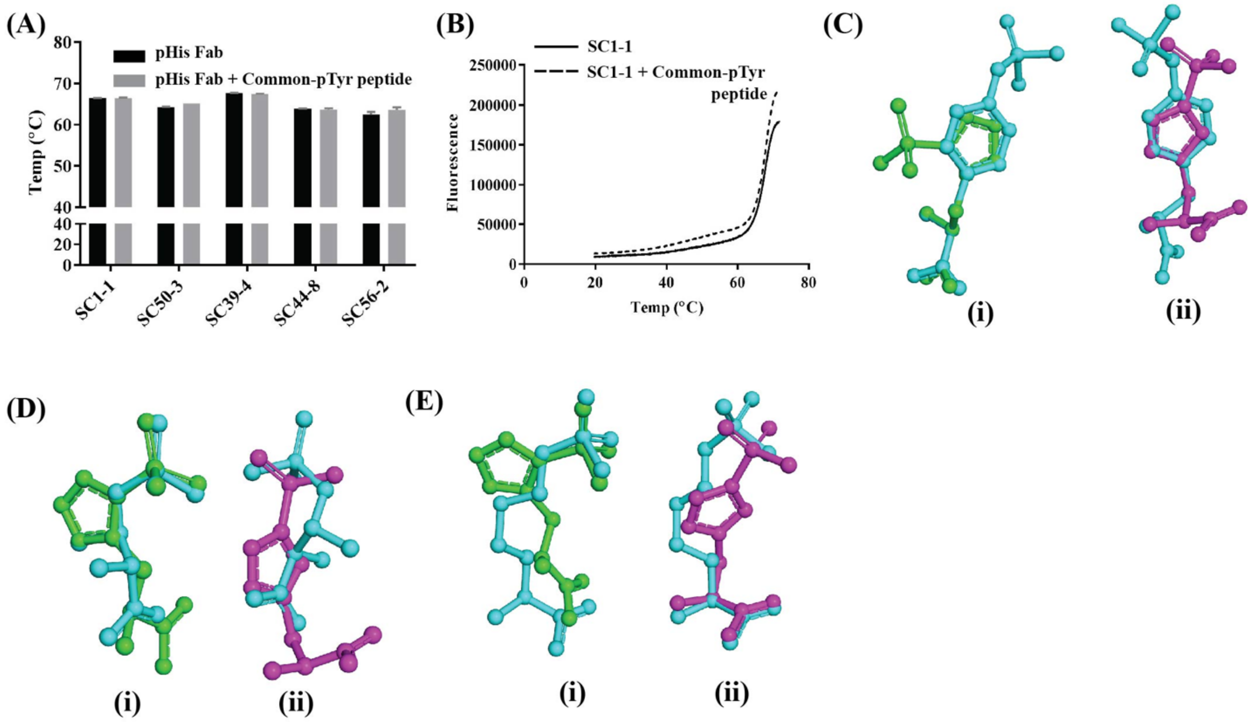

Fig. S11. pHis Fabs non cross-reactivity to the other phosphoamino acid modifications. *(A)* The DSF analysis of pHis Fabs in presence of the pTyr peptide shows that the T_m of the antibodies do not change indicating minimum to no cross reactivity to pTyr modification. *(B)* Representative thermal melt plot of SC1-1 in presence and absence of pTyr peptide. A 3-D structure-based ligand alignment using the LS-Align program- 1-pHis (green) (i) and 3-pHis (magenta) (ii) overlay on cyan colored pTyr *(C)*, pThr *(D)* and pLys *(E)*.

Supplementary tables

Table S1. X-ray data collection and refinement statistics

	**SC1-1**	**SC50-3**	**SC39-4**	**SC44-8**	**SC56-2**
**Ligand**	**RNII-1-pTza-GSDS**	**RNII-1-pTza-GSDS**	**AGAG-3-pTza-AGAG**	**AGAG-3-pTza-AGAG**	**AGAG-3-pTza-AGAG**
**Data collection**					
Beamline	APS 23-ID-D	APS 23-ID-D	ALS 5.0.1	SSRL 12-2	APS 23-ID-D
Wavelength (Å)	1.03315	1.03315	0.97741	0.97946	1.03320
Resolution (Å)[a]	46.7-1.65	49.6-2.34	46.8-1.64	43.8-2.11	47.9-1.95
Space group	P2	$P2_12_12_1$	$P2_12_12_1$	$P2_1$	$P4_12_12$
Unit cell (Å)<br>(°)	86.8, 72.3, 156.7<br>90°, 95.8°, 90°	89.8, 199.7, 204.9<br>90°, 90°, 90°	67.1, 86.1, 167.3<br>90°, 90°, 90°	50.4, 66.3, 58.6<br>90°, 94.9°, 90°	121.1, 121.1, 78<br>90°, 90°, 90°
Total reflections	1,388,099	2,009,178	524,299	69,747	258,589
Unique reflections	230,655	155,051	116,971	21,391	42,281
Multiplicity	6.0(5.1)	13.0(11.2)	4.5(4.4)	3.3 (2.8)	6.1 (6.2)
Completeness (%)	100.0(99.5)	100.0(100.0)	99.1(98.0)	95.8 (79.2)	99.1 (99.8)
Mean (I)/ σ_I	14.5(1.0)	15.7(1.3)	13.3(1.1)	7.6 (1.6)	10.6 (1.1)
R_{merge}[b](%)	11.6(130)	18.1(169)	8.8(151)	18.5 (83.5)	15.2 (129)
R_{meas}[c](%)	12.6(144)	18.9(177)	10.0(173)	22.2 (106)	16.5 (141)
R_{pim}[d](%)	5.0(61.3)	5.2(52.6)	4.6(80.5)	12.1 (64.3)	6.3 (53.8)
$CC_{1/2}$[e](%)	85.6(37.4)	85.8(34.4)	88.6(49.2)	93.2 (32.0)	77.9 (30.6)
**Refinement**					
Refinement resolution (Å)[a]	46.7-1.65	49.6-2.34	46.8-1.64	43.8-2.11	47.9- 1.95
# reflections in refinement (work/free)	219,191/11,408	154,787/7,567	116,780/5,819	20,413/963	40,140/2,104
R_{work}/R_{free} (%)	19.9/22.6	24.4/26.9	18.9/22.5	18.5/23.8	20.0/24.1
# atoms (Fab/Peptide/Solvent)	14,256/160/1027	19,546/292/215	6,432/54/1122	3,198/27/153	3,213/18/354
RMS (bonds)	0.007	0.003	0.022	0.0139	0.0065
RMS (angles)	0.95	0.68	2.14	1.95	0.95
Ramachandran favoured/allowed/ outliers (%)	97.67/2.27/0.06	97.44/2.09/0.46	97.6/2.3/0.1	96.9/3.1/0	97.2/2.8/0
Clashscore[f]	2.8	3.2	1.7	3.0	4.24
Wilson B ($Å^2$)	20	42	20.1	21	27
Average B ($Å^2$) for all atoms/Fab/ Peptide/Solvent	26/26.0/35.4/30.3	54/49/66/37	26/24/25/37	24/23/30/26	36/35/63/46

[a]Numbers in parentheses are for highest resolution shell

[b]$R_{merge} = \Sigma_{hkl} \Sigma_{i=1,n}|I_i(hkl) - \langle I(hkl)\rangle| / \Sigma_{hkl} \Sigma_{i=1,n} I_i(hkl)$

[c]$R_{meas} = \Sigma_{hkl} \sqrt{(n/n-1)} \Sigma_{i=1,n}|I_i(hkl) - \langle I(hkl)\rangle| / \Sigma_{hkl} \Sigma_{i=1,n} I_i(hkl)$

[d]$R_{pim} = \Sigma_{hkl} \sqrt{(1/n-1)} \Sigma_{i=1,n}|I_i(hkl) - \langle I(hkl)\rangle| / \Sigma_{hkl} \Sigma_{i=1,n} I_i(hkl)$

[e]$CC_{1/2}$ = Pearson Correlation Coefficient between two random half datasets

[f]Number of unfavorable all-atom steric overlaps ≥ 0.4Å per 1000 atoms

Table S2. Sequences of the peptides used in this study

No.	Peptide name	Peptide sequence
1	NM23-1-pTza	Arg-Asn-Ile-Ile-1-pTza-Gly-Ser-Asp-Ser
2	ACLYana-1-pTza	Ala-Gly-Ala-Gly-1-pTza-Ala-Gly-Ala-Gly
3	ACLYana-3-pTza	Ala-Gly-Ala-Gly-3-pTza-Ala-Gly-Ala-Gly
4	ACLYana	Ala-Gly-Ala-Gly-His-Ala-Gly-Ala-Gly
5	ACLY	Val-Gln-Phe-Gly-His-Ala-Gly-Ala-Cys
6	NM23	Arg-Asn-Ile-Ile-His-Gly-Ser-Asp-Ser
7	SCS	Arg-Arg-Asn-Gly-His-Ala-Gly-Ala-Ile
8	Common-His	Gly-Glu-Gln-Leu-His-Asp-Leu-Asn-Ala
9	Common-pTyr	Gly-Glu-Gln-Leu-pTyr-Asp-Leu-Asn-Ala
10	Common-pThr	Gly-Glu-Gln-Leu-pThr-Asp-Leu-Asn-Ala
11	Common-pNLe	Gly-Glu-Gln-Leu-pNLe-Asp-Leu-Asn-Ala
12	Common-Lys	Gly-Glu-Gln-Leu-Lys-Asp-Leu-Asn-Ala

Table S3. Elbow angles[#] of pHis Fabs measured by RBOW

Protomer	Elbow angle (°)
SC1-1 Chain AB	159.1
SC1-1 Chain CD	151.3
SC1-1 Chain EF	150.2
SC1-1 Chain HL	158.4
SC50-3 Chain AB	146.9
SC50-3 Chain CD	145.9
SC50-3 Chain EF	150.7
SC50-3 Chain GI	153.9
SC50-3 Chain JK	146.1
SC50-3 Chain HL	147.1
SC39-4 Chain AB	145.6
SC39-4 Chain HL	144.8
SC44-8 Chain HL	142.3
SC56-2 Chain HL	150.6

[#] Elbow angle is the angle formed by heavy chain (V_H and C_H1) and light chain (V_L and C_L) domains along their pseudo-dyad axes (4).

Table S4. Molecular surface area buried on peptide by Fab binding (Å^2)

	**Asn-2**	**Ile-3**	**Ile-4**	**1-pTza-5**	**Gly-6**	**Ser-7**			Total
SC1-1		49	63.1	149	23	6			290
SC50-3	5.7	53	54	152	20	4			288
		**Ala-3**	**Gly-4**	**3-pTza-5**	**Ala-6**	**Gly-7**	**Ala-8**	**Gly-9**	
SC39-4			14	143	38	21			216
SC44-8		32	31	136	64	50	23	37	372
SC56-2		9	33	152	39	21			254

Table S5. Hydrogen bond interactions between pHis Fabs and bound peptides

pHis Fab	pHis Fab		Peptide		Distance* (Å)	Angle@ (°)
	Residue	Atoms	Residue	Atoms		
SC1-1	$A53_H$	N	Phosphate of pTza	O2	2.79	166.2
	$Y58_H$	OH	Phosphate of pTza	O3	2.65	167.9
	$R95_H$	NH1	Phosphate of pTza	O2	2.79	161.5
	$R95_H$	NH2	Phosphate of pTza	O3	2.9	176.1
	$S94_L$	OG	Phosphate of pTza	O4	2.62	130.2
	$S100A_H$	OG	Triazolyl ring	NE2	2.67	102.6#
	$Y91_L$	OH	Triazolyl ring	ND2	3.5	97.3#
	$Y91_L$	OH	1-pTza-5	N	3.38	155.0#
	$S94_L$	N	Ile-3	O	2.67	169.6
SC50-3	$A53_H$	N	Phosphate of pTza	O2	2.76	158.1
	$Y58_H$	OH	Phosphate of pTza	O3	2.45	154.6
	$R95_H$	NH1	Phosphate of pTza	O3	3.16	165.7
	$R95_H$	NH2	Phosphate of pTza	O2	2.75	157.9
	$S94_L$	OG	Phosphate of pTza	O4	2.6	176.1
	$S100A_H$	OG	Triazolyl ring	NE2	2.88	105.3#
	$Y91_L$	OH	Triazolyl ring	ND2	3.48	99.6#
	$Y91_L$	OH	1-pTza-5	N	3.43	157.4#
	$Y28_L$	OH	Asp-8	O	3.8	107.0
	$S94_L$	N	Ile-3	O	2.92	171.8
SC39-4	$N33_H$	ND2	Phosphate of pTza	O4	2.85	170.1
	$W50_H$	NE1	Phosphate of pTza	O4	3.01	157.9
	$S95_H$	OG	Phosphate of pTza	O4	2.59	165.6
	$D97_H$	N	Phosphate of pTza	O1	2.81	154.5
	$G98_H$	N	Phosphate of pTza	O3	3.15	174.5
	$N91_L$	ND2	Phosphate of pTza	O3	2.9	154.6

	$N91_L$	O	Triazolyl ring	NG	3.43	171.2[#]
	$N94_L$	ND2	Gly-4	O	3.0	166.4
	$N94_L$	N	3-pTza-5	O	2.88	155.2
	$R95_L$	NH2	Ala-6	O	2.94	134.1
SC44-8	$K94_H$	NZ	Phosphate of pTza	O4	2.7	141.3
	$G96_H$	N	Phosphate of pTza	O4	3.05	139.4
	$S97_H$	N	Phosphate of pTza	O3	2.99	140.0
	$S97_H$	OG	Phosphate of pTza	O3	2.64	171.1
	$G98_H$	N	Phosphate of pTza	O1	3.07	171.2
	$N99_H$	ND2	Phosphate of pTza	O1	2.62	166.2
	$Y96_L$	OH	Phosphate of pTza	O3	2.72	169.6
	$Y50_H$	OH	Triazolyl ring	NG	3.29	132[#]
	$N99_H$	ND2	Triazolyl ring	NE1	3.4	108.0
	$H91_L$	NE2	Triazolyl ring	NG	3.8	111.5
	$R56_H$	NH2	Gly-7	O	3.6	131.2
	$N99_H$	ND2	Gly-4	O	3.43	154.5
	$W28_L$	NE1	Ala-3	O	2.89	155.5
	$N32_L$	ND2	Gly-4	O	2.98	153.5
	$H91_L$	NE2	Ala-6	N	3.02	112.8[#]
	$S94_L$	O	Gly-7	N	2.97	166.3
	$E95_L$	O	Gly-9	N	2.67	133.2
SC56-2	$K94_H$	NZ	Phosphate of pTza	O3	2.92	160.3
	$G96_H$	N	Phosphate of pTza	O3	3.0	145.0
	$S97_H$	N	Phosphate of pTza	O1	2.84	152.8
	$V98_H$	N	Phosphate of pTza	O4	3.08	178.7
	$S99_H$	N	Phosphate of pTza	O4	2.93	159.9
	$S99_H$	OG	Phosphate of pTza	O4	2.88	152.4
	$Y96_L$	OH	Phosphate of pTza	O1	2.77	163.8
	$Y50_H$	OH	Triazolyl ring	NG	3.39	137.8[#]
	$S99_H$	OG	Triazolyl ring	NE1	3.21	154.5[#]

	$H52_H$	NE2	3-pTza-5	O	2.96	156.6
	$Y91_L$	OH	3-pTza-5	O	2.75	146.4
	$N32_L$	ND2	Gly-4	O	3.0	163.7

[*] Hydrogen bond distance between donor and acceptor atoms < 3.9 Å

[@] >90.0° for the angle made by donor, hydrogen and acceptor atoms

[#] >90.0° for the angle made by donor, acceptor and acceptor antecedent atoms or angle made by hydrogen, acceptor and acceptor antecedent atoms.

Table S6. Classification of CDRs of pHis Fabs by PyIgClassify

	CDRH1	CDRH2	CDRH3	CDRL1	CDRL2	CDRL3
SC1-1	H1-13-4	H2-9-1	H3-14-4	L1-13-2	L2-8-1	L3-12-1
SC50-3	H1-13-4	H2-9-1	H3-14-4	L1-13-2	L2-8-1	L3-12-1
SC39-4	H1-13-1	H2-9-1	H3-9-2	L1-11-1	L2-8-1	L3-13-1
SC44-8	H1-13-1	H2-9-1	H3-11-2	L1-13-2	L2-8-1	L3-13-1
SC56-2	H1-13-1	H2-9-1	H3-11-2	L1-13-2	L2-8-1	Not defined

Table S7. Kinetic data of pHis Fabs with pHis peptides

Fab	ACLY	NM23	SCS	Common-His
SC50-3	K_D: 1.94 x 10^{-7} ± 2.68 x 10^{-9}	K_D: 3.62 x 10^{-7} ± 4.38 x 10^{-9}	K_D: 4.11 x 10^{-7} ± 5.4 x 10^{-9}	K_D: 3.36 x 10^{-7} ± 4.22 x 10^{-9}
	k_a: 1.27 x 10^{5} ± 1.47 x 10^{3}	k_a: 4.85 x 10^{4} ± 4.71 x 10^{2}	k_a: 3.58 x 10^{4} ± 3.73 x 10^{2}	k_a: 7.22 x 10^{4} ± 7.74 x 10^{2}
	k_d: 2.47 x 10^{-2} ± 1.88 x 10^{-4}	k_d: 1.76 x 10^{-2} ± 1.26 x 10^{-4}	k_d: 1.47 x 10^{-2} ± 1.17 x 10^{-4}	k_d: 2.43 x 10^{-2} ± 1.58 x 10^{-4}
SC39-4	K_D: 1.42 x 10^{-6} ± 4.62 x 10^{-8}	K_D: 2.35 x 10^{-6} ± 4.62 x 10^{-8}	K_D: 2.32 x 10^{-6} ± 6.31 x 10^{-8}	K_D: 3.29 x 10^{-6} ± 1.09 x 10^{-7}
	k_a: 7.22 x 10^{4} ± 7.74 x 10^{2}	k_a: 5.14 x 10^{4} ± 1.54 x 10^{3}	k_a: 2.24 x 10^{4} ± 5.32 x 10^{2}	k_a: 1.38 x 10^{4} ± 3.93 x 10^{2}
	k_d: 2.43 x 10^{-2} ± 1.58 x 10^{-4}	k_d: 7.3 x 10^{-2} ± 9.47 x 10^{-4}	k_d: 5.21 x 10^{-2} ± 6.9 x 10^{-4}	k_d: 4.54 x 10^{-2} ± 7.81 x 10^{-4}
SC44-8	K_D: 3.48 x 10^{-7} ± 7.07 x 10^{-9}	K_D: No binding	K_D: 4.67 x 10^{-7} ± 3.57 x 10^{-9}	K_D: No binding
	k_a: 2.8 x 10^{4} ± 4.49 x 10^{2}	k_a: No binding	k_a: 2.93 x 10^{4} ± 1.95 x 10^{2}	k_a: No binding
	k_d: 9.72 x10^{-3} ± 1.2 x 10^{-4}	k_d: No binding	k_d: 1.37 x 10^{-2} ± 5.18 x 10^{-5}	k_d: No binding
SC56-2	K_D: 3.01 x 10^{-6} ± 8.08 x 10^{-8}	K_D: 5.7 x 10^{-6} ± 1.86 x 10^{-7}	K_D: 6.2 x 10^{-6} ± 2.15 x 10^{-7}	K_D: 3.6 x 10^{-6} ± 1.4 x 10^{-7}
	k_a: 5.01 x 10^{4} ± 1.24 x 10^{3}	k_a: 2.22 x 10^{4} ± 6.6 x 10^{2}	k_a: 2.2 x 10^{4} ± 7.1 x 10^{2}	k_a: 3.56 x 10^{4} ± 1.24 x 10^{3}
	k_d: 1.5 x 10^{-1} ± 1.6 x 10^{-3}	k_d: 1.26 x 10^{-1} ± 1.7 x 10^{-3}	k_d: 1.36 x 10^{-1} ± 1.81 x 10^{-3}	k_d: 1.27 x 10^{-1} ± 2.19 x 10^{-3}

Table S8. Applications of pHis antibodies

System under study	Application using pHis antibodies	Reference
KCa3.1 ion channel	3-pHis mAbs were used to show that pHis358 in the KCa3.1 ion channel is a 3-pHis isoform and is essential for relief from copper-mediated inhibition thus allowing increased calcium flux into $CD4^+$ T cells enhancing cytokine production.	(5)
NME1 in Triple negative breast cancer	1-pHis mAbs were used to demonstrate the relationship between the phosphorylated *versus* non-phosphorylated NME and its enzymatic activity in regulation of tumor cell motility in triple-negative breast cancer cell lines	(6)
FAK in esophageal squamous cell carcinoma (ESCC)	pHis mAbs were used to show that pHis58 in FAK is associated with glucose-induced, growth factor–independent proliferation (GFIP) and resistance to therapies that target growth factor signaling.	(7)
NAMPT from NAD^+ salvage pathway	1-pHis antibodies were used to study the effect of the nicotinamide phosphoribosyltransferase (NAMPT) activator on the enzymatic activity of NAMPT, which is involved in cell signaling, DNA repair, cell division and epigenetics in mammalian cells.	(8)
mTOR-driven hepatocellular carcinoma	The pHis mAbs were used to study the role of deregulated histidine phosphorylation in tumorigenesis in an HCC mouse model. Elevated levels of the NME1 and NME2 histidine kinases, and decreased levels of the LHPP pHis phosphatase, correlated with increased tumor severity and reduced overall survival, thus establishing LHPP as the novel tumor suppressor gene	(9)
CusS/CusR two component system in E. coli	Autophosphorylation assay with the pHis mAbs established kinetics and assigned the site of phosphorylation to the N1 position on H271 on CusS.	(10)

Table S9. Crystallization conditions

pHis Fab	Crystallization condition	Cryoprotectant
SC1-1	35 % 2-methyl-2,4-pentanediol<br>0.1 M MES pH 6.29<br>0.2 M Lithium sulfate	Well solution
SC50-3	1.8 M Ammonium sulfate<br>0.1 M Sodium citrate pH 5.5	30 % Ethylene glycol
SC39-4	0.1 M Sodium acetate pH 4.5<br>20 % PEG3000	30 % Ethylene glycol
SC44-8	0.1 M Bicine pH 9.0<br>30 % PEG6000	25 % PEG200
SC56-2	0.1 M Sodium cacodylate pH 6.5<br>1 M Sodium citrate	30 % PEG200

Supplementary references

1. S. R. Fuhs *et al.*, Monoclonal 1- and 3-phosphohistidine antibodies: New tools to study histidine phosphorylation. *Cell* **162**, 198-210 (2015).
2. Y. F. Wei, H. R. Matthews, Identification of phosphohistidine in proteins and purification of protein-histidine kinases. *Methods Enzymol.* **200**, 388-414 (1991).
3. I. K. McDonald, J. M. Thornton, Satisfying hydrogen bonding potential in proteins. *J. Mol. Biol.* **238**, 777-793 (1994).
4. R. L. Stanfield, A. Zemla, I. A. Wilson, B. Rupp, Antibody elbow angles are influenced by their light chain class. *J. Mol. Biol.* **357**, 1566-1574 (2006).
5. S. Srivastava *et al.*, Histidine phosphorylation relieves copper inhibition in the mammalian potassium channel KCa3.1. *eLife* **5**, e16093 (2016).
6. I. Khan, P. S. Steeg, The relationship of NM23 (NME) metastasis suppressor histidine phosphorylation to its nucleoside diphosphate kinase, histidine protein kinase and motility suppression activities. *Oncotarget* **9**, 10185-10202 (2018).
7. J. Zhang *et al.*, Glucose drives growth factor-independent esophageal cancer proliferation via phosphohistidine-focal adhesion kinase signaling. *Cell Mol. Gastroenterol. Hepatol.* **8**, 37-60 (2019).
8. S. J. Gardell *et al.*, Boosting NAD^+ with a small molecule that activates NAMPT. *Nat. Commun.* **10**, 3241 (2019).
9. S. K. Hindupur *et al.*, The protein histidine phosphatase LHPP is a tumour suppressor. *Nature* **555**, 678-682 (2018).
10. T. Affandi, M. M. McEvoy, Mechanism of metal ion-induced activation of a two-component sensor kinase. *Biochem. J.* **476**, 115-135 (2019).
